# Exfoliation procedure-dependent optical properties of solution deposited MoS_2_ films

**DOI:** 10.1038/s41699-023-00376-2

**Published:** 2023-02-24

**Authors:** Robert T. Busch, Lirong Sun, Drake Austin, Jie Jiang, Paige Miesle, Michael A. Susner, Benjamin S. Conner, Ali Jawaid, Shannon T. Becks, Krishnamurthy Mahalingam, Michael A. Velez, Riccardo Torsi, Joshua A. Robinson, Rahul Rao, Nicholas R. Glavin, Richard A. Vaia, Ruth Pachter, W. Joshua Kennedy, Jonathan P. Vernon, Peter R. Stevenson

**Affiliations:** 1 Materials and Manufacturing Directorate, Air Force Research Laboratory, Wright-Patterson Air Force Base, Dayton, OH 45433 USA; 2 UES, Inc., 4401 Dayton Xenia Road, Dayton, OH 45432 USA; 3 Azimuth Corporation, 2970 Presidential Drive, Suite 200, Beavercreek, OH 45324 USA; 4 National Research Council, 500 Fifth St. N.W., Washington, DC 20001 USA; 5 Sensors Directorate, Air Force Research Laboratory, Wright-Patterson Air Force Base, Dayton, OH 45433 USA; 6 Department of Materials Science and Engineering, Materials Research Institute, Center for Atomically Thin Multifunctional Coatings, The Pennsylvania State University, University Park, PA 16802 USA; 7 Department of Chemistry, Department of Physics, Center for Atomically Thin Multifunctional Coatings, The Pennsylvania State University, University Park, PA 16802 USA

**Keywords:** Optics and photonics, Materials for optics, Applied physics, Materials chemistry

## Abstract

The development of high-precision large-area optical coatings and devices comprising low-dimensional materials hinges on scalable solution-based manufacturability with control over exfoliation procedure-dependent effects. As such, it is critical to understand the influence of technique-induced transition metal dichalcogenide (TMDC) optical properties that impact the design, performance, and integration of advanced optical coatings and devices. Here, we examine the optical properties of semiconducting MoS_2_ films from the exfoliation formulations of four prominent approaches: solvent-mediated exfoliation, chemical exfoliation with phase reconversion, redox exfoliation, and native redox exfoliation. The resulting MoS_2_ films exhibit distinct refractive indices (*n*), extinction coefficients (*k*), dielectric functions (ε_1_ and ε_2_), and absorption coefficients (α). For example, a large index contrast of Δ*n* ≈ 2.3 is observed. These exfoliation procedures and related chemistries produce different exfoliated flake dimensions, chemical impurities, carrier doping, and lattice strain that influence the resulting optical properties. First-principles calculations further confirm the impact of lattice defects and doping characteristics on MoS_2_ optical properties. Overall, incomplete phase reconfiguration (from 1T to mixed crystalline 2H and amorphous phases), lattice vacancies, intraflake strain, and Mo oxidation largely contribute to the observed differences in the reported MoS_2_ optical properties. These findings highlight the need for controlled technique-induced effects as well as the opportunity for continued development of, and improvement to, liquid phase exfoliation methodologies. Such chemical and processing-induced effects present compelling routes to engineer exfoliated TMDC optical properties toward the development of next-generation high-performance mirrors, narrow bandpass filters, and wavelength-tailored absorbers.

## Introduction

The development of optical coatings and devices using low-dimensional materials depends on scalable manufacturability with greater control over unavoidable exfoliation technique-induced effects. Two-dimensional (2D) transition metal dichalcogenides (TMDCs) are amenable to large scale liquid phase exfoliation and exhibit distinct quantum optical properties compared to bulk analogs (i.e., bulk optical properties being observed at ≥10 layers^1^). Exemplary 2D TMDC optical interactions include excitonic effects^2–5^, exciton quantum confinement^1,6,7^, charge transfer effects^8^, electric field screening^9^, quantum nonlinear effects^10–13^, and dopant-induced screening offering tailorable optical properties^14^. These representative phenomena constitute fundamental implications toward the pending design and development of low-dimensional high-performance nanophotonic devices such as perfect absorbers, tunable mirrors, and single-photon emitters or detectors. The realization of devices that leverage the low-dimensional optical phenomena of exfoliated few-to-monolayer TMDCs first requires insight surrounding the influence of the selected liquid phase processing chemistry employed.

Procedure-dependent conditions may cumulatively be impacted by the starting bulk material source^15,16^, solvent effects^17,18^, surfactants^19,20^, intercalants^21,22^, exfoliation or stabilization species^23,24^, and surface functionalization^25,26^. Procedure-dependent conditions are further impacted by the respective chemistry, kinetics, mechanics, and solution post-processing associated with the selected exfoliation technique^27–29^. In this work, we experimentally and theoretically investigate the influence of four exfoliation methods on the optical properties of thin films prepared from colloidally stable few-to-monolayer flake dispersions from chemical vapor transport (CVT) grown bulk MoS_2_ powder. Using the derived procedure-dependent optical constants (*n* and *k*), the performance of quarter-wave optical stacks are modeled (i.e., exfoliated MoS_2_ as the high-index layers and a polymer as the low-index layers). The modeled optical responses illustrate the significant impact different exfoliation techniques have on coating design strategies involving exfoliated MoS_2_. Understanding the dependencies between the exfoliation technique and resulting optical properties is necessary in order to design and develop pending low-dimensional optical device applications.

The variation in optical properties reported here demonstrates significant structure-property-processing relationships that depend on the given exfoliation chemistry. The selected technique will determine the resulting exfoliated MoS_2_ crystalline phase^30,31^, flake dimensionality and stacking^1,6^, defect density^32,33^, carrier doping and lattice strain^34–36^, and chemical composition (e.g., residual colloidal reaction by-products or stabilization species)^37,38^. These representative conditions cumulatively impact the excitonic properties of the semiconducting MoS_2_, resulting in direct changes to the optical behavior observed^7,39^. As such, we compare the flake thickness and lateral size dimension, composition, carrier doping, and lattice strain of the exfoliated MoS_2_ product to better understand the optical properties of solution deposited thin films. Perhaps most appealing, these representative exfoliation techniques illustrate the potential to select and modify chemical processing conditions in order to tailor the optical response of films fabricated from exfoliated TMDC solutions, while taking into account the complexity and cost associated with incorporating these methods at scale in manufacturing processes. Our survey of exfoliation technique-induced optical properties of solution deposited MoS_2_ films helps to illustrate the need for greater synthetic control over (for example but not limited to) defect density, lattice phase distributions, intraflake strain, carrier doping, and the amount of residual exfoliation by-products and/or stabilization species. Continued maturation of TMDC exfoliation methods is expected to offer an even broader optical 2D material platform from which to strategically design and develop next-generation low-dimensional material optical coatings and devices.

## Results and discussion

### Exfoliation overview and CVT MoS_2_ source powder

**Fig. 1:**
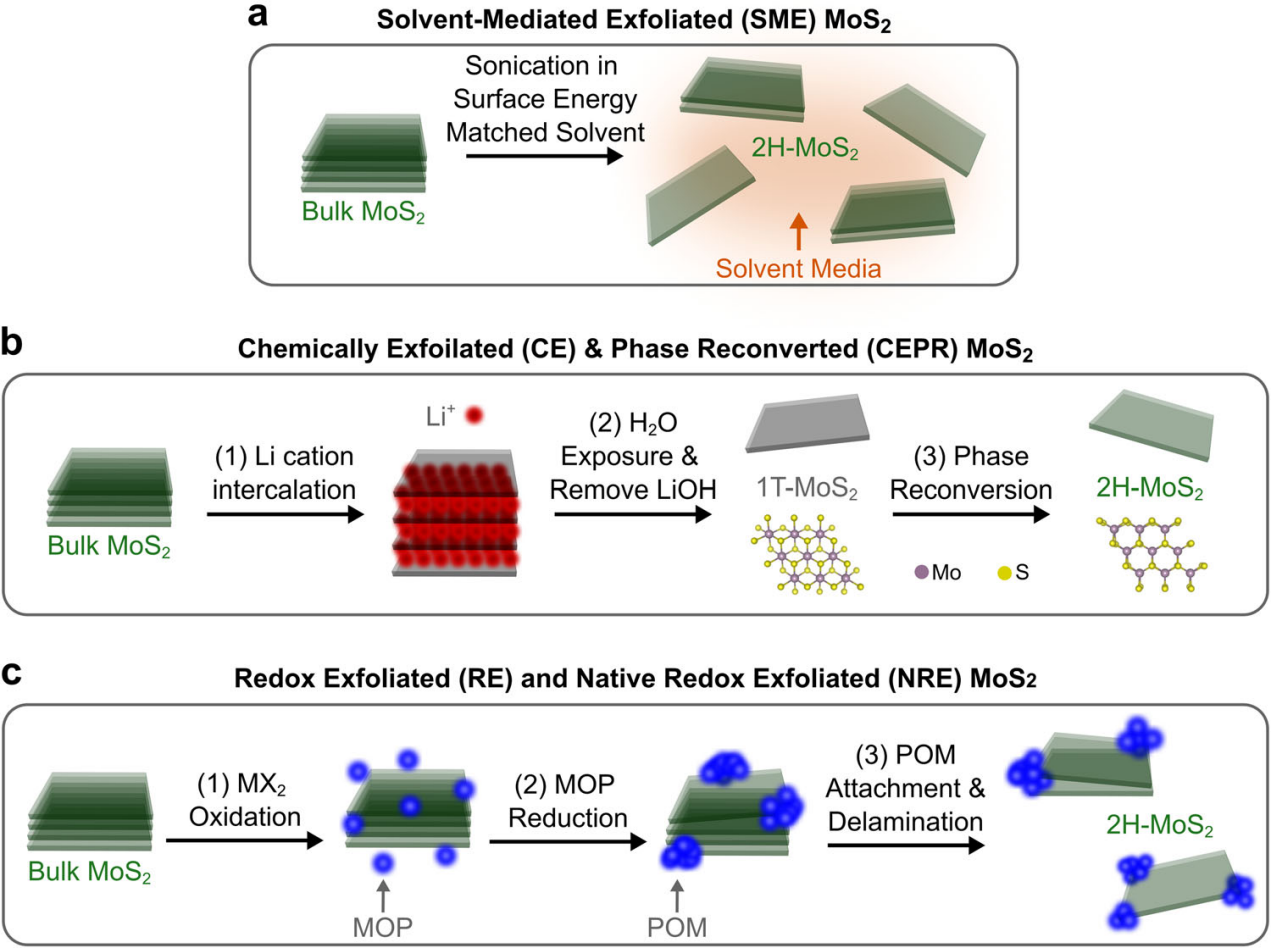
Exemplary liquid phase exfoliation methods used to prepare thin films. Schematics are shown for (**a**) SME MoS_2_, (**b**) CEPR MoS_2_, and (**c**) RE and NRE MoS_2_. Note that (**c**) shows the generalized scheme for RE MoS_2_. In comparison, NRE MoS_2_ is prepared without synthetic oxidation and reduction reagents (e.g., such as with cumene hydroperoxide and hydroquinone, respectively) and represents the first step in (**c**) with native CVT MoS_2_ powder conditions. NRE MoS_2_ is considered part of the redox exfoliation methodology due to the formation of native MOP species. These four exfoliation methods produce the four different procedure-dependent semiconducting exfoliated MoS_2_ assessed herein.

We first provide an overview of the different exfoliation methods used to generate colloidally stable MoS_2_ solution dispersions for thin film deposition (see Fig. 1). These approaches were chosen and formulated to maximize the differences in structure and composition of the exfoliated MoS_2_ products in solution. Unless otherwise stated, the exfoliated MoS_2_ is present in the 2H crystallographic phase. Selection of colloidally stable few-to-monolayer flakes was performed by adjusting centrifugation conditions for the product by monitoring the position and strength of the ~669 nm A exciton peak from solution UV-vis spectrophotometry. Note that all the exfoliation techniques discussed here utilized the same CVT bulk MoS_2_ powder considered to have subtle differences from typical commercial powders used in prior work (see Methods section). The starting material source is an important consideration and prior exfoliation studies have shown variation in few-to-monolayer exfoliated MoS_2_ flake yield and associated photoluminescence^16^. Characterization and chemical impurity content of the CVT MoS_2_ powder is summarized in the sections below, the Methods section, and throughout the Supplementary Information.

### Solvent-mediated exfoliated (SME) MoS_2_

Shown in Fig. 1a, the solvent-mediated exfoliation method was carried out using high-energy mechanical forces in surface energy matched liquid media, N-methyl-2-pyrrolidone (NMP)^40,41^. Here, solvent mediation was used to minimize the addition of surfactant stabilizers. CVT MoS_2_ powder (160 mg) in NMP (10 mL) was probe sonicated with a 13 mm diameter tip at 20% amplitude in an ice bath with a 5 s on/off cycle for 10 h (frequently replenishing the ice bath as needed). After sedimentation of the exfoliated MoS_2_ (15,000 RPM, 10 min), the SME MoS_2_ product was redispersed in a H_2_O/2-propanol mixture to facilitate thin film fabrication without sacrificing colloidal stability. The long processing time, use of high boiling point NMP, and inability to control hydrolysis side reactions (i.e., due to the presence of H_2_O) for this method typically results in small flake sizes, the generation of oxide particles and surface stabilizers, and relatively low yields (~0.2%) of colloidally stable few-to-monolayer MoS_2_^40,42–45^.

### Chemically exfoliated (CE) and chemically exfoliated phase reconverted (CEPR) MoS_2_

MoS_2_ is known to assume a trigonal prismatic coordination (2H with hexagonal symmetry) of the sublattice metal atoms as it is energetically favorable (Fig. 1b). However, the 2H crystal phase can transform into an octahedral coordination (1T with tetragonal symmetry, see Fig. 1b) as well as a less stable distorted coordination (1T’)^31^. During chemical intercalation, the polymorphism of MoS_2_ occurs as a result of a change to the Mo core oxidation state due to electron transfer from the lithiation process. The semimetallic properties of CE 1T-MoS_2_ are compelling (see Supplementary Note 1 and Supplementary Fig. 1), but this crystal phase is known to be metastable under ambient conditions^31,46^. However, CE 1T-MoS_2_ has the potential to be reconverted to the semiconducting 2H phase via subsequent thermal processing in solution. This chemical exfoliation phase reconversion process is unique to MoS_2_ and has not yet been successfully generalized to other TMDCs to-date.

CEPR MoS_2_ was prepared initially following the process described by Eda et al. with lithium intercalation phase change described by Py and Haering to chemically exfoliate 1T-MoS_2_^47,48^. CVT MoS_2_ powder (1.6 g) was added to a round bottom flask and heated to evaporate residual water before vacuum was pulled on the sample. The flask was then purged with argon followed by the addition of hexane (16 mL) and 2.5 M n-butyllithium in hexanes (4 mL). The solution was allowed to stir for ~24 h at 25 °C. During this time, n-butyllithium intercalated between the bulk MoS_2_ layer sheets creating 1T-MoS_2_. A portion of the solution was removed (~2 mL) and centrifuged (10 min at 4000 RPM) to sediment the product for additional processing. The supernatant was discarded and the isolated sediment was resuspended in hexane before centrifuging again to remove residual n-butyllithium. The pellet of 1T Li-MoS_2_ was dried under a nitrogen purge stream for 2 h before adding water (10 mL) and then bath sonicated for ~2 h. Here, the 1T Li-MoS_2_ reacts with H_2_O to generate hydrogen resulting in exfoliation of semimetallic CE 1T-MoS_2_. The product was subsequently washed with additional water over a vacuum filter to further remove residual LiOH.

Conventionally, dried powders of 1T-MoS_2_ have been reconverted to the 2H phase by thermal annealing causing the formation of localized crystalline 2H and amorphous domains. However, prior work suggests phase reconversion can be accomplished in solution by chemical ion exchange at elevated solution temperatures^49,50^. A solution-based phase reconversion approach is potentially advantageous for downstream processing at scale. Here, phase reconversion in solution was performed by precipitating CE 1T-MoS_2_ using NaCl (1 mL, 10 M for 10 mL CE 1T-MoS_2_, settled for 1 h). The supernatant was carefully removed, the precipitate was resuspended in propylene carbonate, and elevated from room temperature to ~200 °C (30 min) over a benchtop hotplate. The resulting solution of CEPR MoS_2_ was centrifuged (15,000 RPM, ~10 min) and the isolated sediment was redispersed in acetonitrile (ACN) for thin film processing.

### Redox exfoliated (RE) MoS_2_

Redox exfoliation is an alternative to the solvent-mediated method, where the inherent hydrolysis and oxidation processes that complement interfacial energy minimization with a high dielectric solvent are stoichiometrically controlled^24,40,44^. This method utilizes water-free conditions, edge-site oxidation pathways, and chemical reduction to form in-situ inorganic metal oxide clusters that can act as colloidal stabilizers (see Fig. 1c). In brief, CVT MoS_2_ powder (300 mg) was treated with a mild oxidizing agent (cumene hydroperoxide, CHP) in an inert polar aprotic solvent to form metal oxide precursors (MOPs). MOP formation was monitored until an equilibrium ion concentration (dependent on surface area of the initial bulk powder agglomerate) was observed using a thiocyanate assay^24,44^. The MoS_2_ and MOP slurry was then treated with a mild reducing agent (hydroquinone) to induce self-assembly of the MOPs into polyoxometalates (POMs). The RE MoS_2_ (1–5% yield) was isolated with centrifugation (15,000 RPM, 10 min), washed three times with ACN, and then redispersed in ACN for thin film processing. Mechanistically, a similar process has been shown to occur in solvent-mediated exfoliation methods when water and/or hydrolytically unstable solvent (e.g., NMP) are probe sonicated for long periods of time. The POMs are thought to provide sufficient coulombic repulsion to overcome the van der Waals forces (which maintain interlayer bulk powder TMDC stacking) resulting in the exfoliation of few-to-monolayer MoS_2_ flakes. It is important to note that the reduction process to form POMs for exfoliation requires careful monitoring of dissolved MOP species and appropriate adjustment to reagent concentration in order to avoid producing larger metal oxide particles. As such, the surface composition of the bulk MoS_2_ crystallites may play a role in the formation of secondary reactions during this redox process.

### Native redox exfoliated (NRE) MoS_2_

The aforementioned SME and RE MoS_2_ methods drive the rate and amount of MoS_2_ exfoliation with the addition of extreme mechanical energy input and/or use of specific hydrolysis, oxidation, and reduction reactions. However, Mo-oxide clusters and mild reductants are native to bulk MoS_2_ powders (whether stored in ambient or inert atmospheres). The presence of these species could directly lead to exfoliation under appropriate solution processing conditions, and without probe sonication or addition of oxidative/reductive reagents. Such is the case for NRE MoS_2_ which begins with CVT MoS_2_ powder (50 mg) added to a polar aprotic solvent (ACN) to form a 5 mg/mL slurry, where trace MOP species were confirmed in solution through colorimetric iodine and molybdenum thiocyanate assays^44^. The slurry was then sealed and placed in a bath sonicator for ~5 h. After this gentle agitation, the solids were allowed to settle for ~5 min before removing the supernatant. NRE MoS_2_ in the supernatant was sedimented (15,000 RPM, 10 min, ~1% yield) and redispersed in ACN for thin film processing.

### Optical properties of exfoliated MoS_2_ thin films

Thin films (~50 nm thick) of exfoliated few-to-monolayer MoS_2_ flakes were deposited on single-side polished C-plane sapphire (from the solution dispersions described above) using high-precision spray coating (see Methods section). Variable angle spectroscopic ellipsometry is used to determine the optical properties of the MoS_2_ films in this work. Ellipsometry permits a comprehensive comparison of the refractive indices (*n*), extinction coefficients (*k*), dielectric functions (*ε*_1_ and *ε*_2_), and absorption coefficients (*α*). These optical properties are derived from optical dispersion data *Ψ* and Δ (as related to the magnitude and phase of the complex reflectivity, *R*, ratio from incident *s*- and *p*-polarized light, $\frac{{R_p}}{{R_s}} = tan\left( {\it{\Psi }} \right)e^{i\Delta }$) using a Lorentz multi-oscillator model for semiconducting MoS_2_ (see Supplementary Note 2)^14,51^. Ellipsometry is ideal as film-to-film optical properties can often be more easily derived independent of concentration, optical density, and radiative transfer effects with appropriate model parameterization (which is not so easily achieved, for example, from UV/vis spectrophotometry)^52–54^. Each MoS_2_ film was characterized using standard ellipsometry methods as opposed to generalized or Mueller matrix ellipsometry methodologies (model parameters are provided in Supplementary Table 1). To confirm this characterization approach, we first determined our MoS_2_ films exhibit no anisotropy and depolarization as a result of comprehensive Mueller matrix spectroscopic ellipsometry (i.e., normalized 15 Mueller elements, see Supplementary Note 3, Supplementary Figs. 2–6 for the C-plane sapphire substrate, and Supplementary Figs. 7–11 for exemplary NRE MoS_2_). For each film, the potential influence of effective medium mixing conditions were assessed in the optical dispersion data analysis. This assessment likewise incorporated a multi-sample analysis to constrain effective medium fit parameters involving MoS_2_ flakes and air, void, or dielectric fraction filling in the film (e.g., linear, Bruggeman, and Maxwell-Garnett mixing methods)^54,55^, as shown in prior work for coalescing monolayer metalorganic chemical vapor deposited MoS_2_ films^56^. However, such assessment involving our MoS_2_ films produced unphysical effective optical dispersions with high mean squared errors (MSEs > 100) and was not included in the optical dispersion data analysis. It is also important to note that effective medium approximations assume consistent chemical composition of the underlying mixed constituents, which is not the case for these exfoliated MoS_2_ colloidal dispersions as dictated by the different chemistries discussed above. As a result, the derived optical properties of the films represent the combined influence of intrinsic material property differences including flake dimensionality, surface adsorbates, chemical composition, lattice doping, and intraflake strain characteristics discussed in the sections below.

**Fig. 2:**
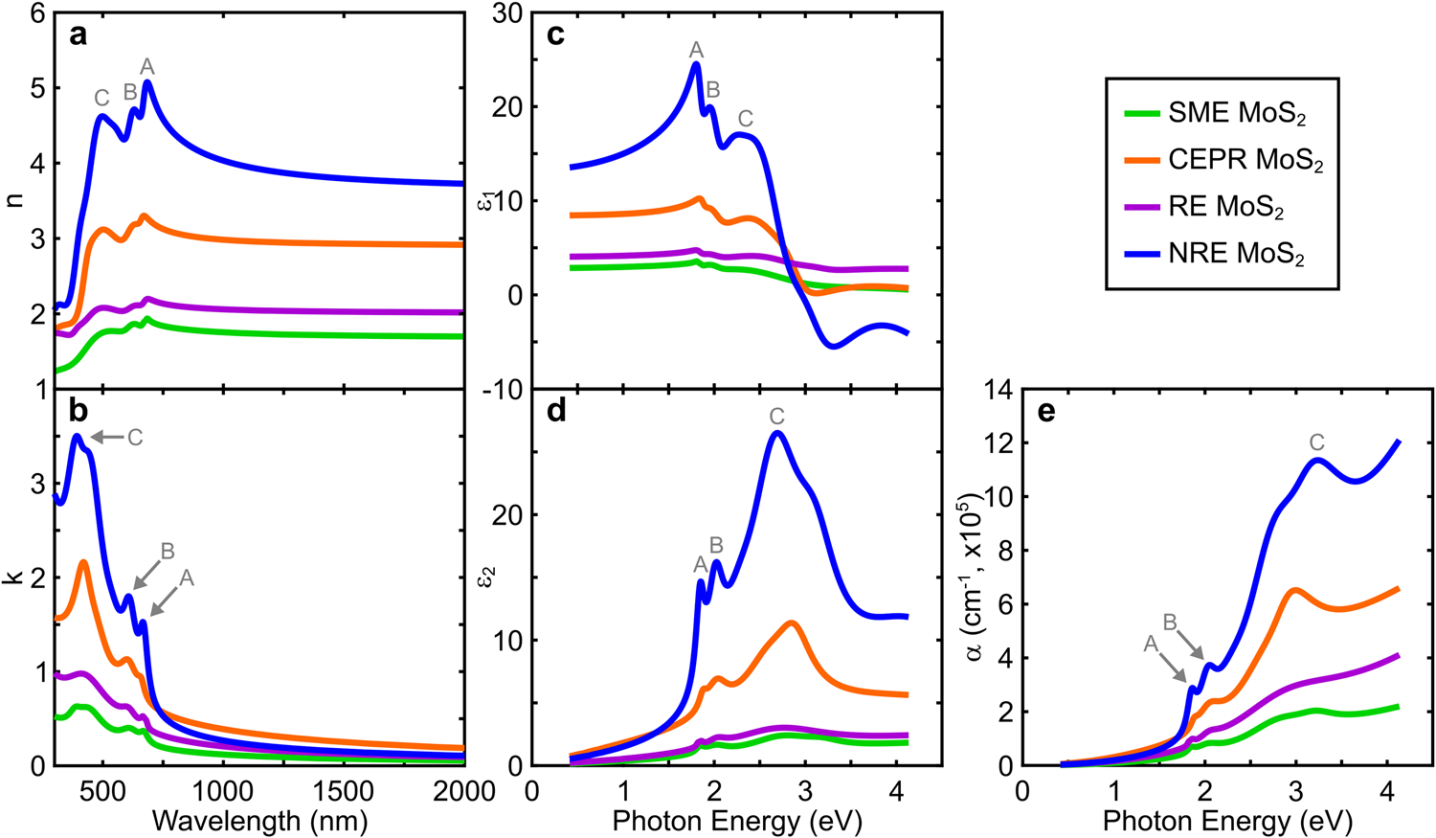
Optical properties of exfoliation procedure-dependent MoS_2_ thin films. **a**, **b** Optical constants, *n* and *k*, **c**,**d** dielectric functions, ε_1_ and ε_2_, and **e** absorption coefficients, α, of MoS_2_ films derived from variable angle spectroscopic ellipsometry optical dispersion data analysis. The A, B, and C excitons are indicated with respect to NRE MoS_2_, which yields the most distinctive optical profile.

Optical constants (*n* and *k*) describe how light is affected as it interacts with a material. Such fundamental properties are critical to support predictive modeling of emergent low-dimensional TMDC optical coating technologies. The refractive indices (*n*) for each MoS_2_ film are shown in Fig. 2a. The extinction coefficients (*k*) are shown in Fig. 2b and define how strongly incident light will attenuate upon entering a material. The change in *n* and *k* observed here is significant. For an arbitrarily selected wavelength of 1000 nm (below the MoS_2_ bandedge), Δ*n* ≈ 2.3 for the most extreme case of NRE MoS_2_ vs. SME MoS_2_ films and ∆*k* ≈ 0.3 for the most extreme case of CEPR MoS_2_ vs. SME MoS_2_ films (∆*k* ≈ 2.9 at the C exciton location in Fig. 2b for NRE MoS_2_ vs. SME MoS_2_ films and ∆*k* ≈ 0.7 below the bandedge for CE 1T-MoS_2_ vs. SME 2H-MoS_2_ films at 1000 nm shown in Supplementary Fig. 1). The observed index contrast is considerable and appealing with respect to optical coating engineering trade space considerations. For comparison, ∆*n* ≈ 0.8 (at 1000 nm) for a conventional high-index contrast all-dielectric optical stack of Nb_2_O_5_ vs. SiO_2_^57^.

The optical responses of semiconducting TMDCs are dominated by the underlying exciton behavior (i.e., Coulombic electron-hole pair interactions)^5,39^. The A, B, and C peak excitons in Fig. 2 are labeled with respect to the NRE MoS_2_ film as it yields the most discernible spectral features. Each MoS_2_ film exhibits spectral exciton characteristics that resemble the A, B, and C excitons as observed for NRE MoS_2_, but the overall magnitude and peak profiles differ considerably—indicative of exfoliation technique-induced effects discussed in the following sections.

The dielectric function (or permittivity) of a material describes the screening of the incident electromagnetic radiation. The complex dielectric function $\left( {\tilde \varepsilon = \varepsilon _1 + i\varepsilon _2} \right)$ is related to the optical constants as *ε*_1_ = *n*^2^−*k*^2^ and *ε*_2_ = 2*nk*. *ε*_1_ is the real part of the dielectric function and describes the degree to which a material can store and remit radiative energy (i.e., the polarizability). This is depicted for the different exfoliated MoS_2_ films in Fig. 2c showing a large range of polarizabilities (e.g., up to Δ*ε*_1_ ≈ 20.9 at 1.807 eV at the A exciton peak). *ε*_2_ is the imaginary part of the dielectric function and describes energy absorption or dissipative dielectric losses due to electronic resonances at optical frequencies. Figure 2d shows a large range of *ε*_2_ responses for the different exfoliation techniques, indicating variable absorption cross-sections of the respective oscillator profiles (e.g., up to Δ*ε*_2_ ≈ 20.5 at 2.9520 eV at the C exciton location). The exfoliated MoS_2_ polarizabilities and dielectric losses observed in Fig. 2c, d further illustrate the potential to engineer optical semiconducting TMDC excitonic behavior as a function of solution formulation and processing conditions.

The absorption coefficient describes the intensity of attenuated incident radiation (or exciton absorption for semiconducting MoS_2_) for the different exfoliation methods and associated thin films. The absorption coefficient (*α*) is expressed as $\alpha = \frac{{4\pi k}}{E}$ as a function of photon energy (E) converted to units of cm^−1^. A low *α* indicates light is poorly absorbed while a high *α* often indicates light is strongly absorbed at a given excitation wavelength. Figure 2e shows a wide range of absorption for the different approaches to exfoliate MoS_2_ (e.g., up to Δ*α* ≈ 9.315 × 10^5^ at 3.2460 eV at the C exciton location). Absorption characteristics can also provide insight toward electron mobility (e.g., poor or uncontrolled light absorption) as well as device performance stability (e.g., temperature or chemical degradation in relation to optical absorption) of MoS_2_ films prepared from exfoliation techniques for optoelectronic device applications^58^. Figure 2e shows the NRE MoS_2_ film exhibits high absorption characteristics suggesting greater electron mobility than CEPR, RE, or SME MoS_2_. Optical absorption behaviors represent fundamentally important material characteristics as the optical properties of MoS_2_ are dictated by the associated excitonic behavior (i.e., exciton optical absorption). As such, cumulative exfoliation technique-induced effects will manifest in such absorption characteristics where high absorption suggests minimal influence and low absorption suggests greater influence.

### Modeled Bragg reflectors

**Fig. 3:**
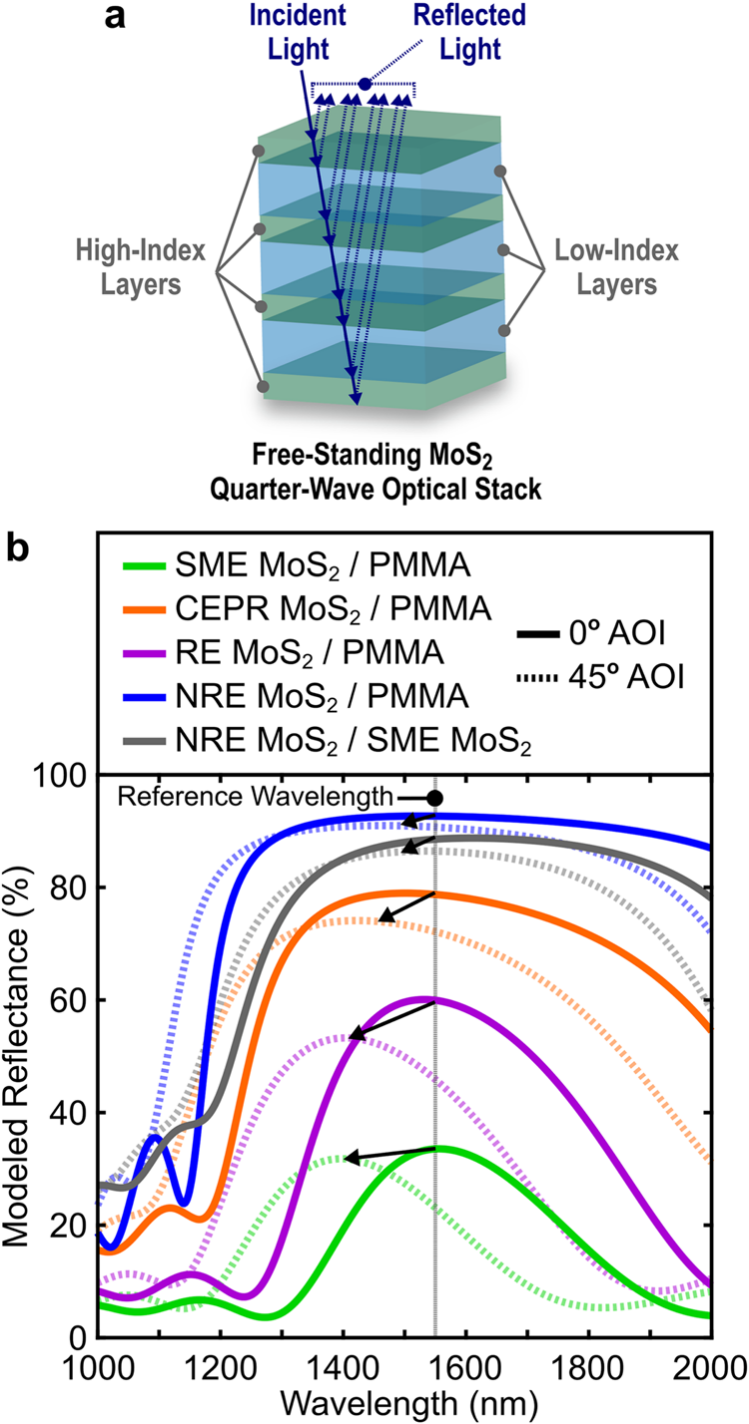
Modeled Bragg reflector spectral performance. Illustrative free-standing MoS_2_ quarter-wave optical stacks with **a** alternating high- and low-index material layers. For the modeled reflectance responses in **b**, the respective exfoliated MoS_2_ type was used for the high-index layer and PMMA for the low-index layer shown in **a**. The modeled percent reflectance for the optical stacks are shown in **b** with a reference wavelength of 1550 nm, a common wavelength in optical telecommunication devices. Also shown are the modeled reflectance responses as a function of angle of incidence (AOI). Solid lines represent the incident light perpendicular, or normal (0°), to the stack surface. Dashed lines represent the incident light at 45° off-normal. The legend indicates the stack high-index layer (left) and the low-index layer (right). This shows that SME MoS_2_ is used as the low-index layer in lieu of PMMA for one example. Most of the differences observed in the modeled reflectance are due to changes in the index contrast (Δ*n*) between the respective high- and low-index layers. Detailed stack coating design parameters are provided in Supplementary Note 4, Supplementary Table 2, and Supplementary Figs. 12, 13.

These observations illustrate a range of optical properties for the exfoliated MoS_2_ thin films prepared from colloidal solutions. While the comparative change in behavior is appealing, the significant procedure-dependent variability observed here has the potential to complicate actual TMDC optical coating or device integration strategies. For instance, we demonstrate the impact of these exfoliation procedure-dependent MoS_2_ optical properties in Fig. 3, which shows the modeled design of optical quarter-wave stacks (Bragg reflectors) using the derived *n* and *k* optical dispersions in Fig. 2a, b. These optical stacks were designed and modeled using conventional transfer matrix methods (see Supplementary Note 4). These designs show how exfoliation procedure-dependent *n* and *k* may limit and potentially augment the design of targeted optical coating performance, for example, at a reference wavelength of 1550 nm. This wavelength is important in optical telecommunications (e.g., the conventional, or C, band is 1530–1565 nm) and was arbitrarily selected to aid the illustration of exfoliation procedure-dependent optical property impact on subsequent optical coating design. For each MoS_2_ technique, the most simplistic layer pair stack was evaluated for each design where the respective MoS_2_ layer is the high-index layer and polymethylmethacrylate, PMMA, was arbitrarily selected for the low-index layer (where *n*_*λ*_ ≈ 1.5). Optical dispersion data for PMMA from Zhang et al. were used in the designs^59^. The most simplistic total layer pair stack configuration was found to be seven layers with the respective high-index MoS_2_ layer on the top and bottom of the optical stack (see Fig. 3a). This means additional layers (and layer pairs) did not improve the modeled reflectance, but removal of layers or layer pairs significantly decreased modeled reflectance (by more than 15%) for each stack design. Layer thicknesses were determined based on the quarter-wave optical thickness of the material with respect to the reference wavelength (see Supplementary Note 4). Air was selected as the entrance and exit media representing a free-standing coating as shown in Fig. 3a. A summary of the coating design parameters is provided in Supplementary Table 2.

The NRE MoS_2_ design yields the greatest modeled reflectance in Fig. 3b due to the higher index contrast (Δ*n*_NREMoS2/PMMA_ ≈ 2.55 at 1550 nm) between the high-index layer (NRE MoS_2_) and the low-index layer (PMMA). SME shows the lowest modeled reflectance with narrower peak widths due to a much lower index contrast between the MoS_2_ and PMMA layers (i.e., Δ*n*_SMEMoS2/PMMA_ ≈ 0.27 at 1550 nm). The illustrative 1550 nm reference wavelength is well below the band edge. As such, differences in the resulting stack designs originate primarily from the magnitude of the index contrast (Δ*n*). This is most obvious when comparing NRE vs. SME or RE MoS_2_ as the high-index layer. Although more subtle compared to the index contrast in these examples, changes in *k* will likewise influence the optical performance behavior by introducing absorption (or anomalous dispersion) in the design. This is observed in Supplementary Fig. 12 where total modeled spectral intensity is shown for each design at normal incidence (i.e., the incident light is oriented perpendicular to the substrate surface or the angle of incidence, AOI, is 0°). Supplementary Fig. 12 shows the difference between peak reflectance and absorptance is low (at 1550 nm) when the Δ*n* between the high- and low-index layer materials is small (e.g., compare Supplementary Fig. 12b, e). We further illustrate the influence of *k* with an all-MoS_2_ Bragg reflector response using NRE MoS_2_ as the high-index layer and SME MoS_2_ as the low-index layer (Δ*n*_NREMoS2/SMEMoS2_ ≈ 2.28 at 1550 nm). This design exhibits similar reflectance characteristics to NRE MoS_2_/PMMA due to the higher index contrast. However, the width of the reflection band for the NRE MoS_2_/SME MoS_2_ response is narrower compared to NRE MoS_2_/PMMA due to the subtle influence of *k* from both MoS_2_ material layers. Lastly, the magnitude of the index contrast will have a significant role in other optical coating performance responses, such as AOI tolerance. For example, Fig. 3b shows changes in the modeled reflectance at the reference wavelength for the incident light at 45° off-normal. SME and RE MoS_2_/PMMA designs have the lowest index contrasts and exhibit a noticeable blueshift in the reflectance peak (for 0° to 45° AOI) along with a ~25% reduction in total reflectance at the reference wavelength. In contrast, the NRE MoS_2_/PMMA and NRE MoS_2_/SME MoS_2_ designs show the greatest tolerance to increasing AOI at the reference wavelength due to the much larger index contrast between the layer materials. Supplementary Fig. 13 provides the total spectral intensity for AOI = 45°. For the high-index contrast examples compared to the low-index contrast examples, these modeled optical coating responses show a ~2.5x increase in reflectance, ~2x increase in bandwidth, and a ~97% reduction in blue shift of peak reflectance at the reference wavelength with increasing incident angle from 0° (normal incidence) to 45° off-normal.

### Morphology and dimension of exfoliated MoS_2_ flakes

**Fig. 4:**
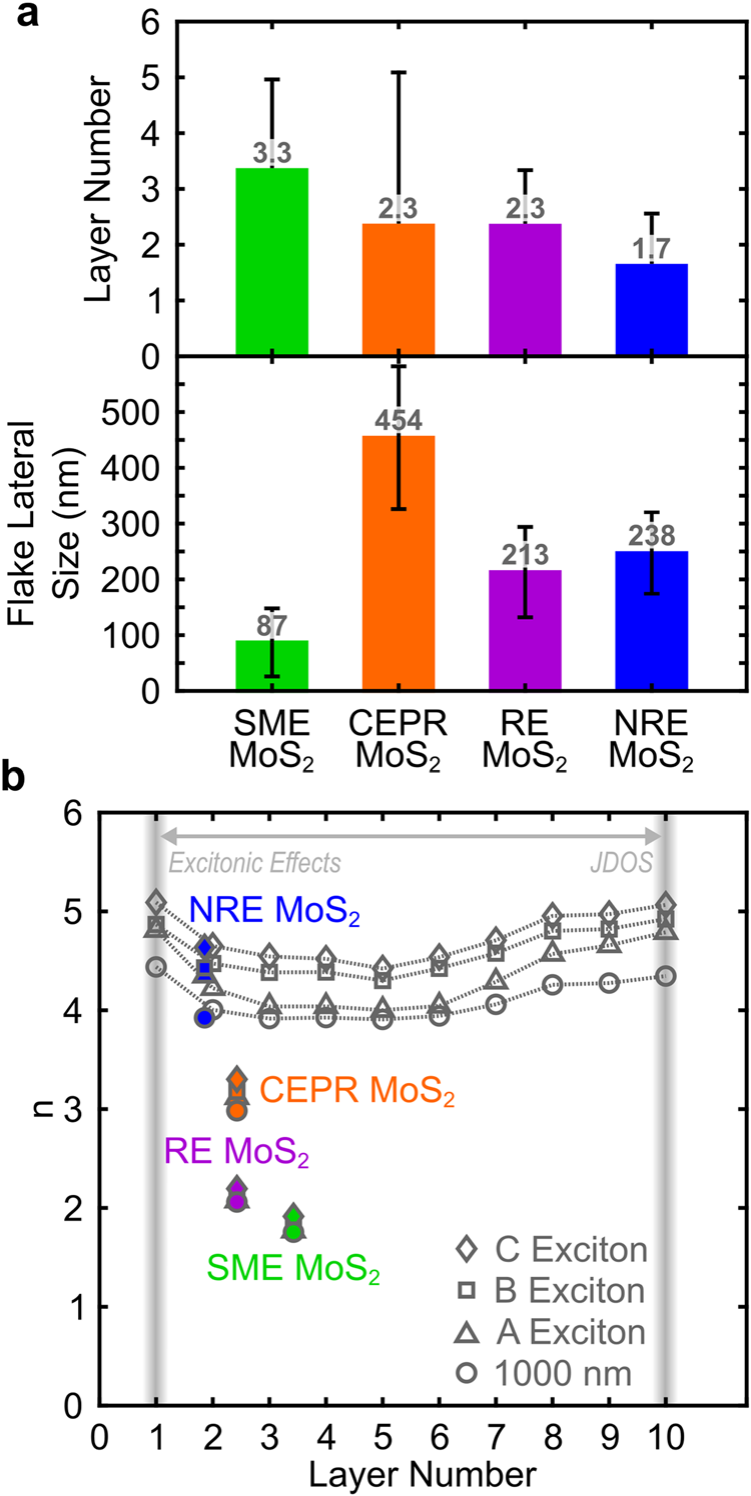
Exfoliation procedure-dependent MoS_2_ dimension. **a** Comparison of average estimated exfoliated MoS_2_ flake dimensions from AFM image analyses with positive standard deviations representing >100 flakes from several images and image areas. Error bars for layer number in **a** correspond to 1.4, 2.5, 0.9, and 0.8 for SME, CEPR, RE, and NRE MoS_2_, respectively. Error bars for flake lateral size in **a** correspond to ±61, ±128, ±81, and ±75 for SME, CEPR, RE, and NRE MoS_2_, respectively. **b** Comparison of derived refractive indices, *n*, at peak A, B, and C exciton energies and at 1000 nm for each MoS_2_ film with respect to estimated exfoliated MoS_2_ flake layer thicknesses. The layer-dependent *n* values reported here are compared to those reported by Yu et al. involving dominating excitonic effects (i.e., layer thickness-dependent quantum confinement interplay) and increase in joint density of states (JDOS) with number of layers^6^. Bulk-like properties are generally considered to occur at ≥10 layers.

A defining morphological characteristic of 2D TMDC flakes or grains is dimensionality and exemplifies the prevailing interest in low-dimensional materials. For example, MoS_2_ exhibits an indirect to direct band gap transition as the number layers in a crystallite reduce to one^60^. Both in and out-of-plane dimensions impact exciton quantum confinement behavior and dielectric field screening effects^4,6,9,14^. Coleman and coworkers have illustrated the ability to predict MoS_2_ size dimension distributions from solution-based optical intensity measurements using assumed edge electronic and thickness-dependent quantum confinement effects^61,62^. Here, we quantify the average and standard deviation of thickness and lateral dimension distributions of the procedure-dependent exfoliated MoS_2_ using atomic force microscopy (AFM, see Methods section) of the few-to-monolayer MoS_2_ from the solution exfoliation formulations described. AFM image analysis of drop cast colloidal solutions give an average exfoliated MoS_2_ flake thickness range of 1.7–3.3 layers and an average lateral size range of 87–454 nm (Fig. 4a). These results are consistent with prior reports that note a substantial impact of exfoliation technique and separation (isolation) procedures on flake dimensions, as discussed by ref. ^16^. SME MoS_2_ exhibits the smallest lateral size distributions consistent with other work and likely results due to the use of high energy mechanical probe sonication input^63^. CEPR MoS_2_ shows fewer layer thickness and the largest lateral size distributions, which is consistent with prior reports for CE MoS_2_^47,48^. RE and NRE MoS_2_ are qualitatively similar in dimension, which is likely given the presumed generalized redox chemistry employed and the use of mild mechanical agitation. The difference between these two methods is likely due to the kinetics of layer delamination in relation to the concentration of inorganic stabilization clusters, where the inorganic clusters are greater for RE MoS_2_. Overall, considering that the product of all these exfoliation methods began with the same starting bulk CVT MoS_2_ source, the range in dimensions is large.

The large Δ*n* = 2.3 of films made from these exfoliation techniques is greater than previous reports that discuss the impact of layer-dependent quantum confinement^6^. For example, Yu et al. experimentally derived layer-dependent dielectric functions of chemical vapor deposited MoS_2_ for dominating excitonic effects at monolayer and near-monolayer thicknesses (i.e., quantum confinement effects) vs. dominating joint density of states with increasing layer thickness^6^. The *n* and *k* derived by Yu et al. are shown in Fig. 4b from 1 to 10 layers (or ~0.7–7 nm). This layer thickness range is comparable to our MoS_2_ films of flakes with variable thickness distributions. Yu et al. report Δ*n* = 0.8 due to changes in numbers of layers, which is substantial. However, this is only a third of what is observed across our exfoliation procedure-dependent MoS_2_ films (Δ*n* = 2.3, Fig. 2). Thus, layer-dependent quantum confined Δ*n* is likely important, but it is not the sole factor influencing the change in magnitude observed in Fig. 2. Figure 4b offers an empirical comparison of our derived refractive indices (at peak exciton wavelengths) as a function of average layer thickness of the colloidally stable MoS_2_ flakes used to generate thin films for characterization. Here, SME, CEPR, and RE MoS_2_ show the greatest deviation from the expected layer-dependent optical properties based on nanoflake thickness alone.

Nanoflake lateral size is another potential consideration—albeit likely having less influence on the optical behavior than flake thickness quantum confinement. Wendumu et al. calculated changes in optical absorption characteristics as a function of lateral flake size (i.e., 3.6–6.5 nm) showing spectral shifts in peak exciton behavior^64^. Complementary experimental work shows similar changes in peak exciton wavelength shifts for lateral nanoscale grain sizes from metalorganic chemical vapor deposited monolayer MoS_2_^56^. The reported lateral dimensions in prior work are substantially less than the average lateral MoS_2_ flake sizes from the exfoliation methods used in this work. As such, lateral flake size likely has minimal influence on the optical properties of our films.

### Chemical composition of exfoliated MoS_2_ flakes

To further understand the optical responses observed in Fig. 2, we quantify the chemical composition of the MoS_2_ films using X-ray photoelectron spectroscopy (XPS). The composition of the colloidally-stable product generated during the exfoliation processes is expected to contribute to the differences observed in the optical properties. For example, a molecularly adsorbed surface layer of ~0.35 nm represents ~50% volume increase over an ideal crystallographic MoS_2_ single layer (~0.7 nm thick). A ~1 nm metal oxide edge passivation of an 80 nm diameter flake represents a ~5% volume increase. Sulfur deficiencies, such as point defects or edge sites, degrade excitonic interactions throughout the MoS_2_ crystalline lattice^65^. Such defects (i.e., vacancies and/or substitutions) dampen exciton peak amplitudes and would potentially shift the system toward greater semimetallic-like behavior. Supplementary Fig. 14 shows measured and fitted Mo 3*d*, Mo 3*p*, and S 2*p* spectra for exfoliated MoS_2_ from each method (see Supplementary Note 5). The binding energy peaks at 229.39 eV and 232.52 eV in Supplementary Fig. 14 originate from Mo^4+^ (MoS_2_), and the higher binding energy peaks at 233.05 eV and 236.18 eV originate from Mo^6+^ (MoO_3_) from the spin doublets Mo 3*d*_5/2_ and Mo 3*d*_3/2_ (with a spin doublet separation of 3.13 eV). The low binding energy peak at 226.6 eV that overlaps with Mo 3*d* is from S 2*s* and corresponds to S^2−^ (from MoS_2_)^66^. Due to the shift in the local bonding environment, 1T Mo^4+^ are typically ~0.8 eV^67^, which can be used to gauge the extent of phase transformation from 1T to 2H during the reconversion process. These assertions were used to fit the spectra in Supplementary Fig. 14. Atomic ratios were calculated from the area under the corresponding peaks^66^. S/Mo and Mo^6+^/Mo (Mo is the sum of Mo^4+^ and Mo^6+^) are used to assess the average chemical composition of the exfoliation products in the films. Additional fit information is provided in Supplementary Table 3.

**Fig. 5:**
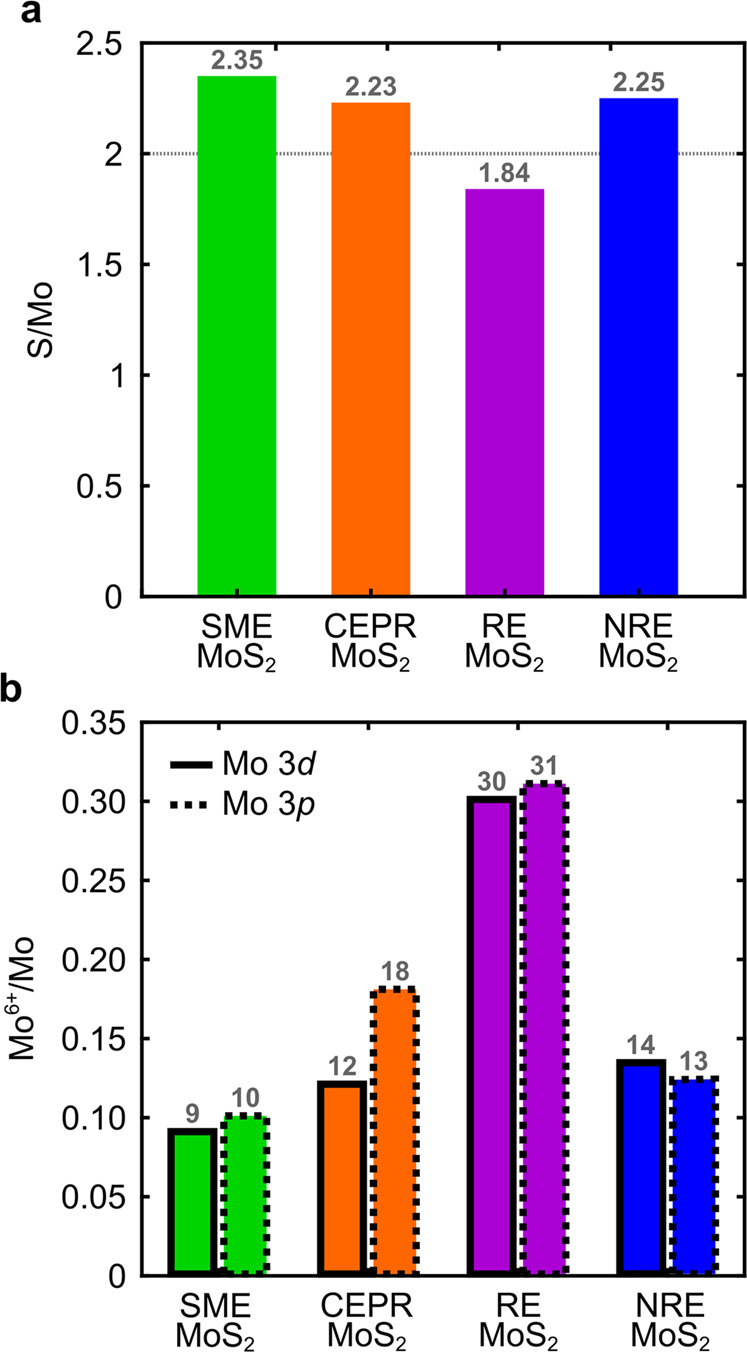
XPS analysis of the exfoliation procedure-dependent MoS_2_ thin films. **a** The S/Mo ratios from the Mo 3*p* and S 2*p* fitted peaks, where stoichiometric and the bulk CVT MoS_2_ are noted with S/Mo = 2 (horizontal dashed line) and 2.54, respectively. **b** The Mo^6+^/Mo atomic percentage from Mo 3*d* and S 2 *s* fitted peaks (solid outline) and from the Mo 3*p* and S 2*p* fitted peaks (dashed outline).

Figure 5a shows the ratio of S/Mo, where Mo is the total molybdenum content. A crystallographic and stoichiometric ideal MoS_2_ material exhibits a S/Mo ratio of 2. The S/Mo ratio for SME, CEPR, and NRE are slightly above 2, but below the S/Mo ratio for the bulk CVT MoS_2_ powder (2.54, Supplementary Table 3, Supplementary Fig. 15, with peak fits for the CVT source powder shown in Supplementary Fig. 16). RE MoS_2_ shows the largest change in S/Mo compared to the source powder. Since all the methods used this source powder, the S/Mo variations reflect changes induced by the specifics of the exfoliation and post-processing procedure employed (i.e., residual stabilization species, Mo-oxides, and by-products not removed in the post-exfoliation wash step). The large difference between RE and NRE MoS_2_ is unexpected given the similar underlying redox exfoliation chemistry. The S/Mo ratio for RE MoS_2_ is likely due to the necessary initial chemical oxidation and reduction used in this exfoliation formulation in response to the surface chemistry of bulk CVT MoS_2_. As such, the greater S/Mo for NRE MoS_2_ is attributed to the absence of this initial chemical oxidation, which is used for RE MoS_2_ to enhance the concentration of soluble Mo-oxide clusters (i.e., MOPs). In comparison, the method to prepare CEPR MoS_2_ does not utilize autoxidation pathways and the greater S/Mo ratio is expected—likely representative of effective post-exfoliation washing of the colloidally-stable product (albeit having unknown by-products throughout the phase reconversion process in propylene carbonate at 200 °C). Furthermore, a single distribution is sufficient to fit the Mo^4+^ peak indicating there is no residual 1T phase present after the reconversion process. Similar S/Mo has been reported for conventional CEPR where phase reconversion induces Mo-oxide species^68^.

Figure 5b summarizes the ratio of Mo^6+^/Mo. The oxidation state of Mo in pure MoS_2_ is +4, whereas oxygen containing species (such as MOPs, POMs, and larger Mo-oxide nanoparticles) will vary up to +6. Thus, Mo^6+^/Mo = 0 for ideal crystallographic MoS_2_, and it will trend toward 1 as chemical species and subsequent defects increase the Mo oxidation state. Such effects may include Mo atoms adjacent to edge sites, S vacancies within the lattice, and/or surface species such as POMs or MoO_3_ nanoparticles. As such, any differences observed from the MoS_2_ films indicate exfoliation procedure-dependent differences to the resulting composition. Mo^6+^/Mo = 0.03 for the bulk CVT MoS_2_ powder (Supplementary Table 3, Supplementary Fig. 15). This implies that the large S/Mo ratio for bulk CVT MoS_2_ powder (2.54) may be due to excess S. Also, for context, previous reports have shown SME MoS_2_ results in flake degradation due to hydrolysis, where the percent oxidate can vary from 5–40% depending on the bulk powder purity, duration of exfoliation, and edge/basal surface ratio^40,69,70^. For RE MoS_2_, previous reports range from 5–12%; however, as noted, this can also greatly increase via specific reaction formulation exfoliation conditions and starting MoS_2_ source powder conditions. Overall, the amount of impurities associated with Mo^6+^ range from ~10–15% for SME, CEPR, and NRE MoS_2_ and approach ~30% for RE MoS_2_. Additionally, 33 at.% oxygen is observed for RE MoS_2_, whereas 7.6, 9.6, and 6.4 at.% is observed for SME, CEPR, and NRE MoS_2_, respectively. These values are generally consistent with prior studies, and the relative trends correspond to the exfoliation processing conditions described above. In particular, the additional chemical oxidation step for RE MoS_2_ likely results in a higher concentration of Mo-oxide species (or MOPs), which then generates greater fractions of MoO_3-x_ complexes upon addition of excess reductant in the exfoliation formulation. Such Mo-oxide complexes are challenging to remove by conventional washing processes^24^. The presence of highly electron withdrawing Mo-oxide species (especially POMs for RE MoS_2_), are known to interact with lattice vacancies and flake edge sites with unterminated Mo and S^24^. The subsequent carrier doping from electron deficient species has been shown to impact optical excitonic characteristics^14^.

Finally, it is important to note that different exfoliation approaches may change the defect concentration within the MoS_2_ layer. This may include changes in edge passivation, S-vacancy formation, and/or creation of intraflake grain boundaries (which the latter is likely for CEPR MoS_2_). These conditions are each known to affect the excitonic behavior for semiconducting MoS_2_. Indeed, prior experimental work has shown significant reduction in *n* and *k* upon mild grain boundary oxidation of metalorganic chemical vapor deposited monolayer MoS_2_^14^. Unfortunately, quantifying the relative amount of defects within the MoS_2_ lattice from the composition and amount of metal oxide (or relevant complex metal oxide species for exfoliated MoS_2_) is not possible with the XPS energy resolution employed.

### Exfoliated MoS_2_ carrier doping and intraflake lattice strain

**Fig. 6:**
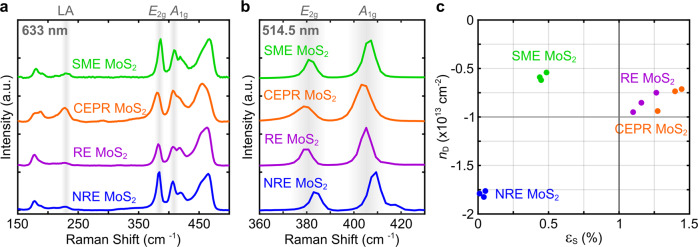
Vibrational modes, carrier doping, and intraflake lattice strain of the exfoliation procedure-dependent MoS_2_. **a** Resonant and **b** non-resonant Raman spectra for each film of exfoliated MoS_2_. **c** Plot of the amount of intraflake doping and strain present in each film representing exfoliated MoS_2_ flakes. The values shown in **c** were calculated with the peak frequencies shown in **b** using Eqs. 1 and 2. Three measurements were taken for the responses shown in **c** to illustrate the potential spread in doping and strain within the same MoS_2_ film.

Raman characterization is also employed to further quantify the differences between the MoS_2_ exfoliation methods. Raman spectra were acquired using 633 nm (resonant) and 514.5 nm (non-resonant) excitation wavelengths for the exfoliated SME, CEPR, RE, and NRE MoS_2_ films and bulk CVT MoS_2_ powder. Under resonant Raman, three peaks are of interest and identified in Fig. 6a: the *E*_2g_, *A*_1g_, and LA modes. The *E*_2g_ and *A*_1g_ modes correspond to in-plane and out-of-plane lattice vibrations, respectively, while the LA mode corresponds to the scattering of longitudinal acoustic (LA) phonons at the M point in the Brillouin zone^71,72^. Additional peaks are observed in Fig. 6a but not discussed in detail due to the complexity of their corresponding symmetry assignments and the resulting difficulty associated with accurate peak fitting^73^. In order to gauge variation in the Raman signal due to potential film non-uniformity, Raman spectra were acquired for three well-separated points on each film.

The intensity ratio of the LA mode with respect to either the *E*_2g_ or *A*_1g_ intensity can be related to the density of lattice defects (or disorder) relative to the crystal size (i.e., a low ratio indicates low disorder and vice versa)^72,74^. First, bulk CVT MoS_2_ powder was analyzed to establish the initial defect density prior to employing the exfoliation methods: the LA/*E*_2g_ ratio is 0.04 (see Supplementary Note 6 and Supplementary Figs. 17, 18), which is similar to chemical vapor deposited monolayers of MoS_2_ having low LA/*E*_2g_ ratios of 0.05–0.07^75^. As such, in agreement with the Mo^6+^/Mo XPS analysis, the CVT MoS_2_ powder used in this study is similar to low-defect chemical vapor deposited MoS_2_ monolayers in terms of basal defect structure. Subsequent increases in the LA/*E*_2g_ modes for the exfoliated MoS_2_ in this work are due to exfoliation technique-induced effects. The average LA/*E*_2g_ peak intensity ratios (Supplementary Fig. 18) for the SME, RE, and NRE MoS_2_ films are 0.08, 0.09, 0.10, respectively—similar to prior studies and slightly higher than the starting powder (Supplementary Fig. 18)^76^. The average ratio for CEPR MoS_2_ is significantly higher at 0.39. This suggests the latter exhibits considerably larger exfoliation technique-induced lattice disorder than the exfoliated MoS_2_ from the other exfoliation techniques. This is consistent with a higher defect concentration due to the polymorphism (i.e., mixed crystalline 2H and amorphous phases) associated with the phase conversion/reconversion methodology for CEPR MoS_2_. Note that lattice rearrangement during reconversion likely does not have a single nucleation site per sheet, but rather occurs at various locations on the flake producing intralayer grain boundaries. This likely results in separate nanoscopic phases, crystallographic orientations, and strain states throughout.

The Raman spectra also provide some insight to the type of Mo-oxide species in the films. All the films show a small peak at 750 cm^−1^ consistent with the *B*_2g_ peak of MoO_2_, with NRE MoS_2_ exhibiting the largest intensity relative to the respective *A*_1g_ peak (Supplementary Fig. 19). This qualitatively suggests NRE MoS_2_ results in more MoO_2_ species than the other exfoliation methods. Combined with XPS, this suggests the Mo-oxide species produced from SME, CEPR, and RE MoS_2_ methods contain more MoO_3_ or MoO_3-x_ by comparison. The origin of these MoO_3-x_ impurities is likely similar between SME and RE MoS_2_ due to the stronger or unregulated chemical oxidation processes used in the associated methods. In contrast, CEPR MoS_2_ may be more strongly influenced by phase reconversion disorder and environmental oxidation of the larger concentration lattice defects (as suggested from the LA/*E*_2g_ peak intensity ratios).

The *E*_2g_ and *A*_1g_ peak frequencies can be related to the amount of strain and carrier doping within the flakes^77,78^. Given the amount of additional peaks present under resonant Raman, non-resonant Raman was used for this purpose to simplify peak-fitting (Fig. 6b). If the MoS_2_ flakes are under an amount of strain (ε_S_) and doped with a carrier concentration (*n*_D_, negative for electrons and positive for holes for responses shown in Fig. 6c), the *E*_2g_ and *A*_1g_ frequencies, *ω*_*E*_ and *ω*_*A*_, will shift expressed as

1 $$\Delta \omega _E = - 2\gamma _E\omega _E^o\varepsilon _s + k_{n,E}n_D$$

2 $$\Delta \omega _A = - 2\gamma _A\omega _A^o\varepsilon _s + k_{n,A}n_D$$

where the Grüneisen parameter (*γ*) and *k*_n_ are constants that relate to changes in the lattice volume and dopant concentration to vibrational frequencies, respectively. *ω*^*o*^ is the frequency corresponding to undoped and unstrained MoS_2_. Using Eq. 1, along with the fitted peak frequencies, the amount of strain and doping in each film can be estimated. For each film, the $\omega _E^o$ and $\omega _A^o$ values appropriate for each exfoliated flake thickness within the respective films were taken from Lee et al.^79^. The respective *γ*_*E*_ and *γ*_*A*_ values used were 0.45 and 0.21 corresponding to few-layer MoS_2_^80^. The respective *k*_*n,E*_ and *k*_*n,A*_ values used were the monolayer values of −0.33 × 10^−13^ and −2.22 × 10^−13^ as multilayer values are not available to our knowledge^77^. As a result of the approximate literature values used in Eqs. 1, 2, the reported strain and doping values shown in Fig. 6c represent comparative trends between exfoliation methods in this work. These results demonstrate NRE MoS_2_ flakes are less p-doped and under less tensile strain than the procedure-dependent exfoliated MoS_2_ flakes within the other respective films. In comparison, SME MoS_2_ is shown to be the most heavily p-doped and under moderately high tensile strain. CEPR MoS_2_ shows the greatest strain, which is consistent with the LA/*E*_2g_ peak intensity ratio likely originating from incomplete 2H phase reconversion (i.e., representing a mix of intraflake crystalline 2H and amorphous phases). RE MoS_2_ appears to be similar to CEPR MoS_2_ in both doping and strain; however, the influence here is expected to be from differences in exfoliation formulation involving the maximized concentration of Mo-oxide (i.e., POMs) that act as p-type dopants with strong electron withdrawing potential. Note that the oxygen content in Mo-oxide lattices is well-known to depend on the chemical environment and synthesis route. For example, MoO_3_ has a very high work function (6.5–7 eV) and is used as an electron injection layer in devices^81^. The work function decreases as the oxygen content decreases to MoO_2_, and the Mo oxidation state takes on mixed values from +6 to +4, respectively. In contrast, POMs are highly electron deficient and act as electron acceptors. Thus, additional chemical analysis is necessary to attribute specific oxygenated Mo species to the degree of MoS_2_ doping for a given exfoliation formulation.

In general, the Raman trends help explain why films formed from NRE MoS_2_ exhibit near-pristine MoS_2_ optical properties compared to the other exfoliation methods examined. Additionally, the potentially higher MoO_3_ vs. MoO_2_ content for SME MoS_2_ compared to NRE MoS_2_ may be one reason for the lower refractive index and extinction coefficient for the former (Fig. 2), despite both having similar overall Mo^6+^/Mo ratios (~10–13%) and atomic oxygen content (~6–7%) shown in Fig. 5. Such influence in compositional difference is further suggested by the increase in both doping and strain for SME MoS_2_, shown in Fig. 6c.

### Perspective on tailoring exfoliated MoS_2_ optical properties

Our experimental assessment of the different exfoliated MoS_2_ in thin films illustrates a broad range of optical property, chemical composition, carrier doping, and strain characteristics. Continued maturation of exemplary chemistry-driven TMDC exfoliation methods and processing is expected to offer even greater tailorability of the technique-induced optical properties of exfoliated MoS_2_. For instance, the chemical exfoliation phase reconversion (for CEPR MoS_2_) and redox exfoliation (for RE MoS_2_) methods represent appealing chemistry-driven approaches with potentially broad in-line processing range for optical property tailorability. In addition to the optical properties shown in Fig. 2, these methods are suggested due to the greater change in doping (0.22 × 10^−13^ cm^−2^ for CEPR MoS_2_ and 0.20 × 10^−13^ cm^−2^ for RE MoS_2_) and strain (0.17% for both CEPR MoS_2_ and RE MoS_2_) compared to the other exfoliated MoS_2_ shown in Fig. 6c. SME MoS_2_ procedure-dependent effects are likely less tailorable due to the processing mechanics involved. In comparison, NRE MoS_2_ from bulk CVT MoS_2_ is appealing due to the lack of additional reagents and overall exfoliation formulation simplicity.

Further optical property control may be accessible via phase reconversion evolution dynamics and compositional POM dopant-induced screening favored by the method to prepare CEPR and RE MoS_2_, respectively. Our synthesis and experimental characterization of CEPR MoS_2_ in this work (building upon previous work^31^) exemplifies the ability to utilize technique-induced phase engineering of exfoliated MoS_2_ to tailor the optical properties. The origin of phase converted and reconverted MoS_2_ polymorphism is attributed to electron transfer to the Mo metal core. Transfer to the Mo core changes the oxidation state resulting in a crystalline phase shift to maintain stability (CE 1T-MoS_2_). After exfoliation (upon the introduction of water to the CE 1T-MoS_2_ material) the structure can be reconverted to 1H/2H-MoS_2_ by displacing the electron from the Mo core through thermal energy input. A greater range of optical properties from differing intraflake phase domains may be achieved through variable thermal processing control and with different chemical ion exchange cations. The redox exfoliation method represents another candidate in-line processing route to tailor the optical properties of MoS_2_. The composition of the POM exfoliation species from artificial redox exfoliation can be altered based on the given starting TMDC bulk powder^24,44^. As a result, electron withdrawing potentials can likely be altered depending on the composition of the POM complex, which may lead to tailorable dopant-induced screening effects. Representative effects are shown in Supplementary Fig. 20 where Mo POM complexes were prepared, isolated, and applied to near-pristine metalorganic chemical vapor deposited MoS_2_ (see Supplementary Note 7). We note that the change in optical properties is relatively small compared to complementary work with organic adsorbates^14^. This may suggest that the difference in magnitude for RE MoS_2_ in Fig. 2 represents necessary in-line processing effects to the basal surface (vs. stepwise physisorption as shown for the responses in Supplementary Fig. 20). A tailorable range of optical properties from in-line dopant-induced screening may be possible with greater control in POM complex composition and morphology.

To gain further insight into the variation of exfoliated MoS_2_ and potential tailorable range of the procedure-dependent optical properties, the effects of sulfur (S) vacancy defects and doping were computationally analyzed. Such effects resemble prominent technique-induced effects of the redox exfoliation method (shown from XPS and Raman characterization) in order to understand the significant change in optical property response between RE and CEPR MoS_2_. We employed the many-body non-self-consistent GW-BSE method (i.e., Green’s function approximation, G, with a screened Coulomb interaction, W, and Bethe Salpeter equation, BSE) as well as density functional theory (DFT) due to computational limitations dependent on the system size, including spin-orbit coupling (see Supplementary Note 8). For the purposes of this work, we consider a single S-vacancy in the MoS_2_ monolayer as a point defect example. Effects of p-doping were subsequently modeled by removing partial electrons from the MoS_2_ monolayer, 0.05e and 0.1e per primitive cell are shown as examples.

**Fig. 7:**
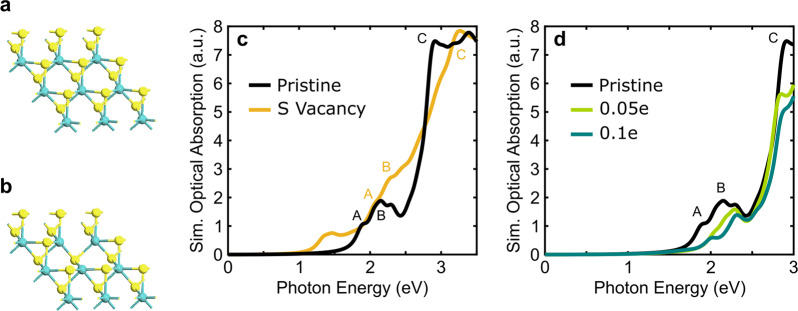
Theoretical calculation of pristine, S-vacancy defect, and p-doped MoS_2_ optical absorption spectra. Representative supercells for **a** pristine MoS_2_ and **b** MoS_2_ with a S-vacancy (cyan = Mo and yellow = S). **c** G_0_W_0_-BSE-SOC optical absorption spectra for pristine monolayer MoS_2_ and having a S-vacancy. **d** G_0_W_0_-BSE-SOC optical absorption spectra for pristine monolayer MoS_2_ upon removing 0.05e electrons and 0.1e electrons—illustrative of generalized p-doping oxidation.

First, we discuss the effect of introducing S-vacancy within the monolayer (the optimized structures are compared in Fig. 7a, b). The calculated optical absorption spectra are shown in Fig. 7c, d. The A, B, and C peak energies of pristine monolayer MoS_2_ at 1.89, 2.15, and 2.91 eV, respectively (see Supplementary Table 4), show agreement with experimental data for the A and B peaks. However, the C peak is underestimated due, in part, to the idealized system and level of theory employed (G_0_W_0_-BSE rather than sc-GW-BSE). Upon inclusion of a S-vacancy, we note broadening of the A and B absorption peaks and the emergence of a localized vacancy exciton at lower energy (0.67 eV below the A peak). In addition, blue-shifts by 0.18, 0.10, and 0.33 eV for the A, B and C peaks, respectively, were calculated. However, note that relative to the experimental systems, we used an unrealistically high defect density due to computational limitations in the G_0_W_0_-BSE calculations, which may lead to inaccuracy. To qualitatively assess the 1.04 × 10^14^ vs. 3.74 × 10^13^/cm^2^ vacancy densities, optical absorption spectra were calculated at the PBE-SOC level for both the smaller and larger supercells (see Supplementary Note 8). By decreasing the defect density, the blue-shift decreases and the vacancy peak intensity is lowered (vacancy peak to bulk B peak intensity ratio is decreased from 0.3 for the defect density of 1.04 × 10^14^ to 0.2 for 3.74 × 10^13^/cm^2^, Supplementary Fig. 21). The vacancy peak is likely difficult to detect experimentally as it may be too weak in realistic samples or overlap with exciton peak broadening due to adsorbed chemical species, doping, or other lattice defects. Still, this observation could explain, in part, why films fabricated from NRE MoS_2_ exhibit optical characteristics that more closely resemble near-pristine, defect-free, fully stoichiometric MoS_2_.

Additionally, Raman spectroscopy indicates that the different exfoliation methods produce products with variable degrees of doping (even within the same film, Fig. 6c). In comparison to pristine monolayer MoS_2_, the corresponding optical absorption spectra for p-doped samples (see green and teal lines in Fig. 7d) at the G_0_W_0_-BSE-SOC level demonstrate the A and B peaks broaden, with some red-shifts observed. The effect is enhanced with an increase in p-doping. By removing 0.1e, the Fermi level (see band structures at the PBE-SOC level in Supplementary Fig. 22) is shifted down and part of the first valence band is less occupied. Thus, optical transitions from the unoccupied part of the first valence band to the first conduction band are forbidden and exciton absorption is suppressed. Note that such effects would vary in practice based on the relative work-function of the surface adsorbed species. A reduction in exciton absorption due to increased p-doping would subsequently suppress the derived optical property spectra, as is partially the case for SME, PR, and RE MoS_2_ qualified by XPS and Raman characterization. While we computationally illustrate the qualitative influence of S-vacancy defects and oxidative p-doping, theoretically discerning the relative contribution of these effects is beyond the scope of this study. However, future complementary experimental-theoretical efforts are expected to further quantify the multiplex exfoliation technique-induced consideration presented in this report and lead to greater improvements in engineered 2D optical exfoliated TMDC materials.

Advances in processing stable solution dispersions of exfoliated TMDCs represent important steps toward low-dimensional materials on an industrial scale. Large-scale liquid phase exfoliated TMDCs with tailorable optical properties (i.e., *n*, *k*, *ε*_1_, *ε*_2_, and *α*) are further expected to enable the development of next-generation high-precision optical devices and coatings. The development of in-line process tailoring of TMDC optical properties is expected to mature and, as discussed herein, may be achieved through the strategic utility of accessible exfoliation chemistries with associated processing conditions—which will ultimately dictate optical device design, integration, and performance. Our experimental assessment of the different exfoliated MoS_2_ methods and thin films (from the same starting source) illustrate a broad range of optical properties, directly correlated to the selected exfoliation procedure. These exfoliation procedures and related chemistries produce different exfoliated flake dimensions, chemical impurities, carrier doping, and lattice strain that cumulatively impact the resulting optical properties. First principles calculations further illustrate that both S-vacancy defects and electron deficiencies (p-doping) also impact the optical properties. These analyses (in conjunction with prior reports) surrounding the influence of relevant procedure-dependent factors on exfoliated MoS_2_ optical properties suggest the following: significant contribution to the reported differences in optical properties are largely due to (1) the procedure-dependent chemical composition of constituents from the MoS_2_ solution formulations in the resulting MoS_2_ film, and (2) the procedure-dependent doping and strain of the MoS_2_ flakes from surface adsorbates and associated phase distribution.

To illustrate the impact of such technique-dependent optical properties on device design strategies and performance, we modeled quarter-wave optical stacks using the derived *n* and *k* from the thin films (i.e., exfoliated MoS_2_ as the high-index layer and PMMA or SME MoS_2_ as the low-index layer for illustration). In comparison to the modeled optical coating response of the lowest index contrast design (SME MoS_2_/PMMA), the highest index contrast stack design (NRE MoS_2_/PMMA) yielded a ~2.5x increase in reflectance, ~2x increase in bandwidth, and a ~97% reduction in blue shift of peak reflectance when changing the incident angle from 0° to 45° off-normal. Considering only Δ*n*, the chemical exfoliation phase reconversion (CEPR MoS_2_) and native redox exfoliation (NRE MoS_2_) methods likely provide the most in-line procedure-dependent tailorability of the optical properties for such designs. Overall, these experimental and theoretical findings suggest compelling routes toward TMDC optical coatings via selected exfoliation chemistries and processing methods (e.g., phase engineering, composition, and doping). For example, modifying the thermal treatment and ion exchange for the CEPR method may afford tuning of the phase reconversion process via the relative amount of amorphous to crystalline 2H MoS_2_ phase distributions, as well as associated intraflake doping and strain characteristics. Alternatively, adding additional surface adsorbates with varying electron withdrawing potentials relative to MoS_2_ may yield dopant-induced screening effects to tailor the optical properties of RE MoS_2_. Such methodologies are expected to expand the design and development of next-generation high-performance mirrors, narrow bandpass filters, wavelength-tailored absorbers, and high quantum efficiency photodetectors utilizing low-dimensional optical materials.

## Methods

### MoS_2_ crystal growth via chemical vapor transport (CVT)

Noncommercial bulk CVT MoS_2_ powder was used in this work. We grew bulk MoS_2_ using a traditional vapor transport synthesis technique involving a two-step process. First, sulfur chunks (Alfa Aesar, 99.9995%) were loaded together with molybdenum foil (Alfa Aesar, 99.95%, 0.025 mm thick) in a 2:1 ratio together with 100 mg of I_2_ crystals (Alfa Aesar, 99.9985%) under vacuum in a 25 cm long and 20 mm wide (2 mm wall thickness) quartz ampoule. The sample was ramped to 800 °C at a rate of 30 °C/h and held there for 96 h. After the sample cooled, it was removed from the furnace and the ampoule opened inside a fume hood. After the I_2_ evaporated, the MoS_2_ precursor was ground and resealed with 100 mg of I_2_ inside a second quartz ampoule. This ampoule was then heated to 1220 °C at 30 °C/h and held for one week until large crystals were visible on the cold end of the tube. The samples were then allowed to cool to extract the crystals for subsequent liquid phase exfoliation shown in Fig. 1.

The starting source powder is an important consideration when assessing the exfoliation procedure-dependent optical properties of MoS_2_ (see Supplementary Note 9). We provide XPS (Supplementary Table 3, Supplementary Fig. 15) and Raman (Supplementary Figs. 17, 18) data of pretreated commercial (Sigma Aldrich, 15 μm powders, lot: WXBC8313V) and as-grown CVT MoS_2_ powders for comparison. This also includes an optical property source powder comparison of thin films made from exfoliated MoS_2_ (Supplementary Fig. 23), scanning electron microscopy and energy dispersive spectroscopy (SEM and EDS, Supplementary Figs. 24, 25), structural characterization via X-ray diffraction (XRD, Supplementary Fig. 26), and high resolution transmission electron microscopy (HRTEM, Supplementary Figs. 27, 28). We note that the bulk CVT MoS_2_ powders used in this work utilize I_2_ as the transport agent during crystal growth. Extra precautions were taken to remove residual I_2_ from the powders before processing them during exfoliation. This was confirmed via XPS, EDS, and UV-vis titrations of supernatents containing bulk powders in CHCl_3_ and ACN, confirming the absence of residual I_2_.

Note that the stability of CVT MoS_2_ is minimal in polar aprotic solvents when using a probe sonicator. In comparison, even with residual vapor transport I_2_ removed from the CVT MoS_2_ (confirmed via XPS and UV-vis titrations of supernatants containing bulk powders in CHCl_3_ and ACN), CVT MoS_2_ powder can be exfoliated using the native redox method in several solvents that have been shown to offer poor colloidal stability due to bulk MoS_2_ crystallite mismatch surface energy^27^. For example, solution dispersions of NRE MoS_2_ were prepared in CHCl_3_, THF, and other non-polar solvents, despite the presumed extreme solubility parameter mismatch. For comparison, commercial MoS_2_ powders (known to have been prepared without I_2_ transport methods) do not exhibit this range in solvation. Furthermore, the addition of I_2_ to a bath sonicated solution does not appear to trigger or enhance the exfoliation rate of MoS_2_ powders grown without the iodine transport agent. As such, we consider the use of I_2_ vapor transport during the growth of CVT MoS_2_ to have a potentially beneficial impact on the crystallite surface energy as compared to other MoS_2_ powders. This represents an important focus of investigation for future exfoliation mechanistic studies.

### High-precision spray coating

Exfoliated MoS_2_ thin films were deposited using a SCS PrecisionCoat Benchtop spray coater with a 2 mm nozzle diameter. Spray coating conditions between depositions were maintained to target a ~50 nm film thickness for each exfoliated MoS_2_ type. The inlet N_2_ pressure for the spray coater was ~20 psi. The syringe pressure was ~10 psi and the atomization nozzle pressure was set to ~2 psi. The needle position was set to ~2 μm and the nozzle to substrate distance was ~15 cm. This configuration resulted in a deposition rate of approximately 1 mL/min for exfoliated MoS_2_ dispersed in anhydrous ACN. The spray deposition was automated for consistency with a movement speed of 100 mm/min performing single passes above the substrate with 5 mm spacings to evenly coat the sapphire substrates.

### Variable angle spectroscopic ellipsometry

A J.A. Woollam RC2 ellipsometer was used to characterize the optical properties of the exfoliated MoS_2_ films. Optical dispersion data were collected from 300–2500 nm at 50–80° angles (5° intervals) for standard ellipsometry. The optical assessment using Mueller matrix ellipsometry is discussed in Supplementary Note 3. Optical dispersion data analysis was performed using CompleteEASE v6.55 (J.A. Woollam). Each respective model incorporated the single-side or double-side polished C-plane sapphire substrate and the respective exfoliated MoS_2_ film.

### Flake thickness and size distribution measurements

Exfoliated MoS_2_ flake thicknesses and size distributions were determined via atomic force microscopy (AFM). Measurements were performed on a Bruker Dimension Icon in soft-tapping mode. Samples were prepared by drop-casting a dilute exfoliated MoS_2_ solution on UV-O treated ultraflat silicon wafer and air drying. Bruker Nanoscope analysis software was utilized for all flake dimension analysis. In most cases, many different images were evaluated per exfoliated MoS_2_ type and >100 flakes were assessed to generate the distribution data shown in Fig. 4.

### X-ray photoelectron spectroscopy (XPS)

A Thermal Scientific ESCALAB Xi^+^ X-ray photoelectron spectroscopy spectrometer microprobe at an analyzer pass energy of 70 eV was used to measure the exfoliated MoS_2_ on sapphire substrates. As such, we obtained survey scans and core level spectra of C 1*s*, O 1*s*, Mo 3*p*, Mo 3*d*, S 2*p*, and Al 2*p* (see Supplementary Fig. 14). The XPS spectra were analyzed using a CasaXPS v.2.3.19 software package. All core level spectra were calibrated and referenced with respect to the adventitious carbon C 1*s* at a binding energy (BE) of 284.8 eV^82^. The chemical compositions of the exfoliated MoS_2_ and associated oxidation were derived from the high-energy scans. The spectra were fitted with Gaussian-Lorentzian line shapes after Shirley background subtraction.

### Raman spectroscopy

Raman spectra were obtained using a Renishaw InVia Raman microscope using 1800 and 1200 lines/mm gratings for the 514.5 and 633 nm excitation lasers, respectively. A 50x objective lens with a numerical aperture of 0.75 was used. Baselines from the spectra were corrected using asymmetrically reweighted penalized least squares smoothing (arPLS) before fitting with pseudo-Voigt line shapes.

### Supplementary information

Supplementary Information

## Data Availability

The data that support this manuscript and Supplementary Information are available from the corresponding author upon reasonable request.
