# Supplementary material for: Exfoliation procedure-dependent optical properties of solution deposited MoS2 films
Source: NPJ 2D Mater Appl. 2023 Feb 24;7(1):12. doi: 10.1038/s41699-023-00376-2 (PMC11041683; doi:10.1038/s41699-023-00376-2)
Supplement: Supplementary file 1 — Supplementary Information [file 41699_2023_376_MOESM1_ESM.pdf]

Supplementary Information

Exfoliation Procedure-Dependent Optical Properties of Solution Deposited MoS_2 Films

Robert T. Busch[1,2], Lirong Sun[1,3], Drake Austin[1,2], Jie Jiang[1], Paige Miesle[1,2], Michael A. Susner[1], Benjamin S. Conner[4,5], Ali Jawaid[1,2], Shannon T. Becks[1,2], Krishnamurthy Mahalingam[1,2], Michael A. Velez[1,2], Riccardo Torsi[6], Joshua A. Robinson[6,7], Rahul Rao[1], Nicholas R. Glavin[1], Richard A. Vaia[1], Ruth Pachter[1], W. Joshua Kennedy[1], Jonathan P. Vernon[1], and Peter R. Stevenson*[,1]

[1]Materials and Manufacturing Directorate, Air Force Research Laboratory, Wright-Patterson Air Force Base, Ohio, 45433, USA

[2]UES, Inc., 4401 Dayton Xenia Road, Dayton, Ohio, 45432, USA

[3]Azimuth Corporation, 2970 Presidential Drive, Suite 200, Beavercreek, Ohio 45324, USA

[4]National Research Council, 500 Fifth St. N.W., Washington D.C., 20001, USA

[5]Sensors Directorate, Air Force Research Laboratory, 2241 Avionics Circle, Wright-Patterson Air Force Base, Ohio, 45433, USA

[6]Department of Materials Science and Engineering, Materials Research Institute, Center for Atomically Thin Multifunctional Coatings, The Pennsylvania State University, University Park, Pennsylvania, 16802, USA

[7]Department of Chemistry, Department of Physics, Center for Atomically Thin Multifunctional Coatings, The Pennsylvania State University, University Park, Pennsylvania, 16802, USA

*Email: peter.stevenson.2@us.af.mil

Table of Contents

Supplementary Note 1. Additional Optical Property Comparison of Exfoliated MoS_2 3

Supplementary Note 2. Spectroscopic Ellipsometry Optical Dispersion Data Analysis: Fit Parameterization.. 5

Supplementary Note 3. Standard vs. Mueller Matrix Spectroscopic Ellipsometry 7

Supplementary Note 4. Modeled Quarter-Wave Optical Stacks 21

Supplementary Note 5. XPS Analysis 25

Supplementary Note 6. Additional Raman Analysis 29

Supplementary Note 7. Impact of POMs on the Optical Properties of Monolayer MOCVD MoS_2 Films 32

Supplementary Note 8. Computational Details.. 36

Supplementary Note 9. Starting Source Powder and Comparison 40

Supplementary References.. 48

Supplementary Note 1. Additional Optical Property Comparison of Exfoliated MoS_2

As will be shown throughout this Supplementary Information, there are exfoliation technique-induced optical properties beyond those reported in the main text. The first illustration of such additional processing-induced optical property changes is demonstrated by the chemically exfoliated (CE) 1T-MoS_2 product before phase reconversion occurs. The semimetallic CE 1T-MoS_2 response in Supplementary Figure 1 is compared to the semiconducting exfoliated MoS_2 types which shows dissimilar spectral characteristics in the visible regime and much greater absorption into the infrared. The phase-dependent optical properties of MoS_2 have been comprehensively discussed in prior work from first principles calculations and experimental characterization[1]. The optical dispersion data analysis for the 1T-MoS_2 differed from those reported in the main text and is discussed in Supplementary Note 2.

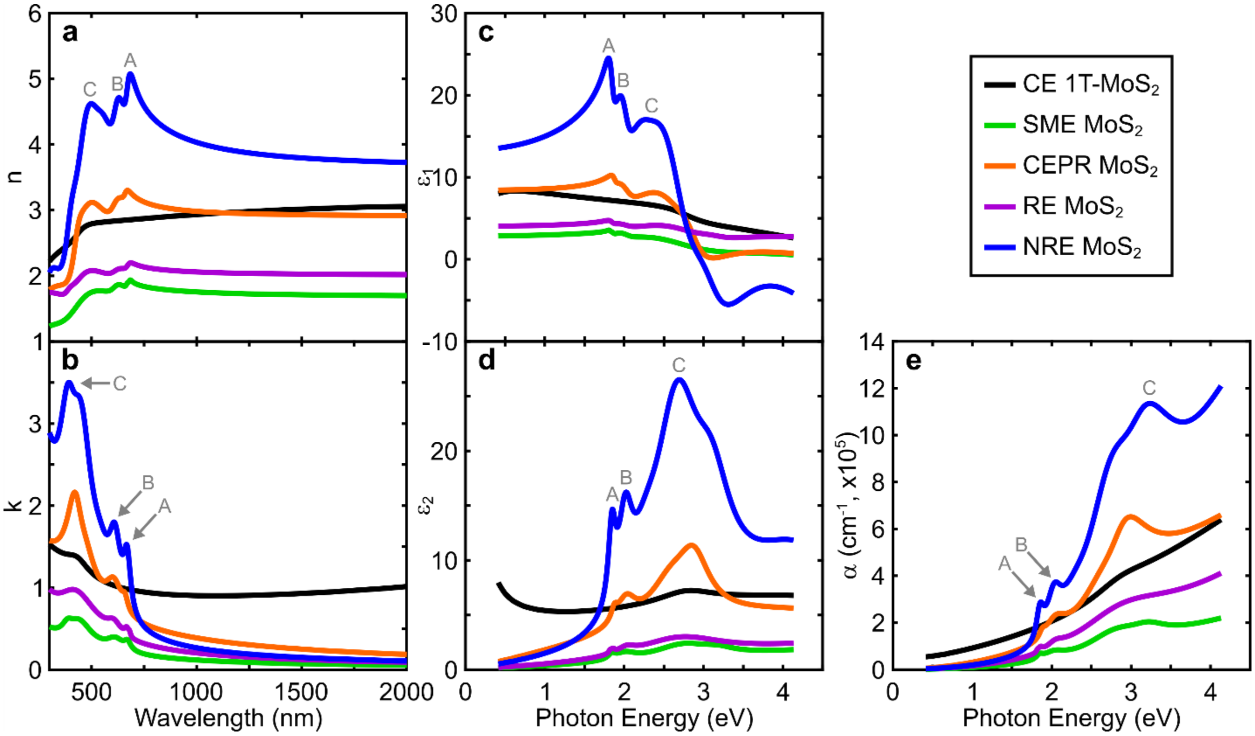

Supplementary Figure 1. Optical properties of exfoliation procedure-dependent MoS_2 thin films including CE 1T-MoS_2. (**a-b**) Optical constants, *n* and *k*, (**c-d**) dielectric functions, ε_1 and ε_2, and (**e**) absorption coefficient, α, of exfoliation procedure-dependent exfoliated MoS_2 (including CE 1T-MoS_2) derived from standard variable angle spectroscopic ellipsometry. The A, B, and C excitons are indicated with respect to NRE MoS_2, which yields the most distinct optical dispersion profile for the exfoliated semiconducting MoS_2.

Supplementary Note 2. Spectroscopic Ellipsometry Optical Dispersion Data Analysis: Fit Parameterization

The complex refractive index ($\tilde{n} = n + ik$ where $\varepsilon_1 = n^2 - k^2$ and $\varepsilon_2 = 2nk$ for the real and imaginary parts of the complex dielectric function, $\tilde{\varepsilon}$, respectively) from the multi-oscillator Lorentz (Supplementary Equation 1) and Drude-Lorentz (Supplementary Equation 2) formalisms used in this work are given by

$$\tilde{\varepsilon}(\omega\ or\ E) = \varepsilon_1 + i\varepsilon_2 = \varepsilon_o - \sum_{k=1}^{N} \frac{f_k}{\omega_k^2 - \omega^2 - i\omega\gamma_k} \quad (1)$$

$$\tilde{\varepsilon}(\omega\ or\ E) = \varepsilon_1 + i\varepsilon_2 = \varepsilon_o - \sum_{k=1}^{N} \frac{f_k}{\omega_k^2 - \omega^2 - i\omega\gamma_k} - \frac{\omega_p^2}{\omega^2 + i\omega\tau_p} \quad (2)$$

where ε_o is the permittivity of free space, f_k is the resonant oscillator amplitude strength, γ_k is the resonant peak oscillator width, and ω_k is the resonant peak oscillator wavelength for the kth oscillator for the Lorentz component. For the Drude component, ω_p is the plasma frequency and τ_p is the damping term (or the effective Drude decay constant). Supplementary Equation 1 and 2 are written as a function of wavelength (ω), but can also be represented as a function of energy (E) as reported in the main text for the dielectric functions and absorption coefficients in Fig. 2.

All exfoliated MoS_2 optical properties reported in the main text used the Lorentz formalism and the multi-oscillator fit parameters are provided in Supplementary Table 1. This formalism was likewise used for the reported optical properties in Supplementary Figure 1, except for CE 1T-MoS_2. CE 1T-MoS_2 is a semimetal material and a Drude-Lorentz model was used for the

ellipsometry optical dispersion data analysis as reported in prior work[1]. The fit parameters for CE $1T\text{-}MoS_2$ are provided in Supplementary Table 1.

Supplementary Table 1. Fit parameters for exfoliated MoS_2.

Lorentz Parameter[a]	Oscillator 1	Oscillator 2	Oscillator 3	Oscillator 4	Oscillator 5	Oscillator 6
CE 1T-MoS_2 (derived film thickness = 57 nm; MSE = 1.99)						
Amp	1.084	0.861	4.951	-	-	-
Br	0.855	37.895	8.855	-	-	-
En	2.812	4.821	5.978	-	-	-
ρ[e]	-	-	-	2.087×10^{-3}	-	-
τ[f]	-	-	-	1.688	-	-
SME MoS_2 (derived film thickness = 53 nm; MSE = 1.59)						
Amp[b]	0.439	0.601	1.298	0.340	1.825	-
Br[c]	0.106	0.326	0.902	0.481	5.418	-
En[d]	1.844	2.017	2.773	3.197	5.747	-
CEPR MoS_2 (derived film thickness = 41 nm; MSE = 1.34)						
Amp	1.931	0.692	4.408	5.365	8.261	3.793
Br	1.474	0.067	0.495	0.749	0.611	1.205
En	1.469	1.867	2.017	2.579	2.886	4.195
RE MoS_2 (derived film thickness = 56 nm; MSE = 1.23)						
Amp	0.197	0.340	0.532	1.831	0.510	1.681
Br	0.037	0.068	0.253	1.384	2.163	4.977
En	1.821	1.857	2.032	2.782	4.919	5.205
NRE MoS_2 (derived film thickness = 51 nm; MSE = 0.96)						
Amp	7.039	7.747	3.378	19.572	8.895	10.027
Br	0.109	0.217	0.421	0.684	0.605	2.024
En	1.847	2.017	2.276	2.686	3.117	4.318

[a]The variables shown here are those used in the computational software. They are related to Supplementary Equation 1 in the as follows: [b]Amp is unitless amplitude where $f = Amp \cdot Br \cdot En$ in units of eV^2. [c]Br is the broadness of the peak where $\gamma = Br$ in units of eV. [d]En is the energy of the peak in units of eV. Note that Supplementary Equation 1 is as a function of wavelength (ω). Equation S2 is used for CE $1T\text{-}MoS_2$ as it is a semimetal. [e]ρ is DC resistivity in units of Ohm·cm. [f]τ is the dampening term or scattering time in units of fs.

Supplementary Note 3. Standard vs. Mueller Matrix Spectroscopic Ellipsometry

Standard ellipsometry was used to derive the optical properties shown in Fig. 2 of the main text. This implies incident *p*- and *s*-polarized light is reflected as *p*- and *s*-polarized light (i.e., negligible polarization, cross-polarization, depolarization, or retardance occurs). To confirm the efficacy of our approach, standard ellipsometry and Mueller matrix ellipsometry were compared. Mueller matrix ellipsometry can be a powerful optical characterization technique to identify potential influence from film anisotropy (e.g., uniaxial or biaxial anisotropic materials in relation to contributing surface roughness, index gradation, or effective medium effects) and depolarization resulting from morphological film non-uniformity, instrument bandwidth artifacts, or angular spread effects. For a given material, measuring every Mueller element can be redundant; however, Mueller matrix ellipsometry is the only way to truly quantify anisotropic depolarization if present in a film (i.e., the quadratic depolarization index, P_D)[2].

Depolarization is a likely concern when characterizing films made from exfoliated materials where film morphology may be influenced by the size, shape, and ordering distributions of few-to-monolayer thick flakes. Depolarization is likewise a requisite parameterization factor when employing effective medium approximations[3]. If no appreciable anisotropy or depolarization is present (as is common for most thin films, <100 nm), the resulting optical dispersion data analysis will simply yield an isotropic response (since $n_x = n_y = n_z$) and can be appropriately modeled as such. It is important to note that an anisotropic material can very rarely be fit with an isotropic model. Pseudo-anisotropic materials or substrates are an exception to this and occur due to incomplete in-plane orientation-dependent characterization about the optical axis of a material (i.e., the need to employ Euler angle ϕ, θ, and Ψ rotation characterization). This is the case for C-

plane sapphire shown in Supplementary Figure 2-5 where $n_x = n_y \neq n_z$ or $n_o \neq n_e$, being the ordinary (n_o) and extraordinary (n_e) refractive indices, respectively. Overall, if a film is anisotropic with dominating morphological contribution, it must be modeled as such or any attempt to analyze the optical dispersion data will be unsuccessful (meaning unphysical predicted responses, very high MSEs, and no derived film property assessment).

We begin our optical property assessment by first illustrating the response for C-plane sapphire, which is known to exhibit subtle uniaxial anisotropy. However, note that no such effects are observed from reflectance spectroscopic ellipsometry in Supplementary Figure 2 (standard) and Supplementary Figure 3 (Mueller matrix). This is because the measured responses originate from the substrate surface. However, the effects of uniaxial anisotropy for C-plane sapphire can be observed under transmission spectroscopic ellipsometry (compare m_{23}, m_{24}, m_{32}, m_{42} in Supplementary Figure 4). Transmission spectroscopic ellipsometry is valuable as it is more sensitive to in- and out-of-plane index differences and sample orientation, if present in a film. In contrast, reflection ellipsometry can yield greater sensitivity to in-plane anisotropic index responses, but it is typically insensitive to out-of-plane index responses and sample orientation dependencies, if present in a film. As such, in order to truly evaluate the presence of optical anisotropy, it is necessary to employ both reflectance and transmission spectroscopic anisotropy. This is what was done here for the C-plane sapphire substrates used in this work. The resulting derived ordinary and extraordinary optical constants are shown in Supplementary Figure 5 (representing a miscut of the C-plane sapphire substrate of ~0.19° by the manufacturer). The model for the ordinary and extraordinary optical constants were subsequently used as the substrate layer when modeling the exfoliated MoS_2 films.

Percent depolarization and the P_D for the sapphire substrate are shown in Supplementary Figure 5 and 6, respectively. These plots show there is no depolarization from the substrate under standard reflectance spectroscopic ellipsometry and some when evaluating the P_D. The P_D observed in Supplementary Figure 6 is due to the uniaxial anisotropy of the substrate (fit using both reflectance and transmission spectroscopic ellipsometry data). Although there is some depolarization observed in the P_D, the effects can be addressed in our anisotropic parameterization to improve the confidence level of the derived optical constants.

Using the NRE MoS_2 film as a representative example for all the exfoliated MoS_2 films, the standard reflectance analysis is shown in Supplementary Figure 7 and the reflectance-based Mueller matrix analysis is shown in Supplementary Figure 8. From the Mueller matrix analysis in Supplementary Figure 8, no cross-polarization is observed in the off-diagonal elements that would be indicative of in-plane anisotropy (m_{13}, m_{14}, m_{23}, m_{24}, m_{31}, m_{32}, m_{41}, m_{42}). In addition, no retardance (m_{13}, m_{14}) or polarizance (m_{31}, m_{41}) is observed, which are common spectral signatures for anisotropic films or substrates. As such, this suggests there is no in-plane anisotropy for the NRE MoS_2 film. To confirm, transmission spectroscopic ellipsometry is shown in Supplementary Figure 9 for NRE MoS_2. Here, we observe spectral differences in the UV/vis regime due to the exciton peaks in the diagonal elements compared to the sapphire substrate. In the off-diagonal elements, we observe subtle spectral characteristics that resemble those of the C-plane sapphire substrate. As a result, the fit shown in Supplementary Figure 9 is performed using only the uniaxial anisotropy from the substrate—meaning there is no out-of-plane anisotropy for the exemplary NRE MoS_2 film. Although no anisotropic parameterization was necessary in the optical dispersion analysis for NRE MoS_2 (and all the other exfoliated MoS_2 films characterized in this work), we show the derived optical constants when conventional spectroscopic ellipsometry data is appended

with transmission spectroscopic ellipsometry data in Supplementary Figure 10. For simplicity, we used the standard reflectance spectroscopic ellipsometry throughout this work for each exfoliated MoS_2 film. This modeling approach is further confirmed by the depolarization assessment.

Percent depolarization and the P_D for the NRE MoS_2 film is depicted in Supplementary Figure 11, showing negligible depolarization (i.e., being identical to the C-plane substrate in Supplementary Figure 6). This implies there are no measurement artifacts originating from the exfoliated MoS_2 film that would complicate our optical dispersion analysis. Such artifacts could arise from film morphological differences (e.g., inconsistent layering of the exfoliated MoS_2 flakes in the film or voids in the film) or film nonuniformity that would cause excessive scattering of the incident light. For the films used in this work, we consider the experimentally derived optical properties shown here to be the product of the process-dependent exfoliated MoS_2 material (e.g., overall defect density, chemical composition, and ensemble flake dimensionality) as dictated by the associated exfoliation chemistry, kinetics, mechanics, and post-processing.

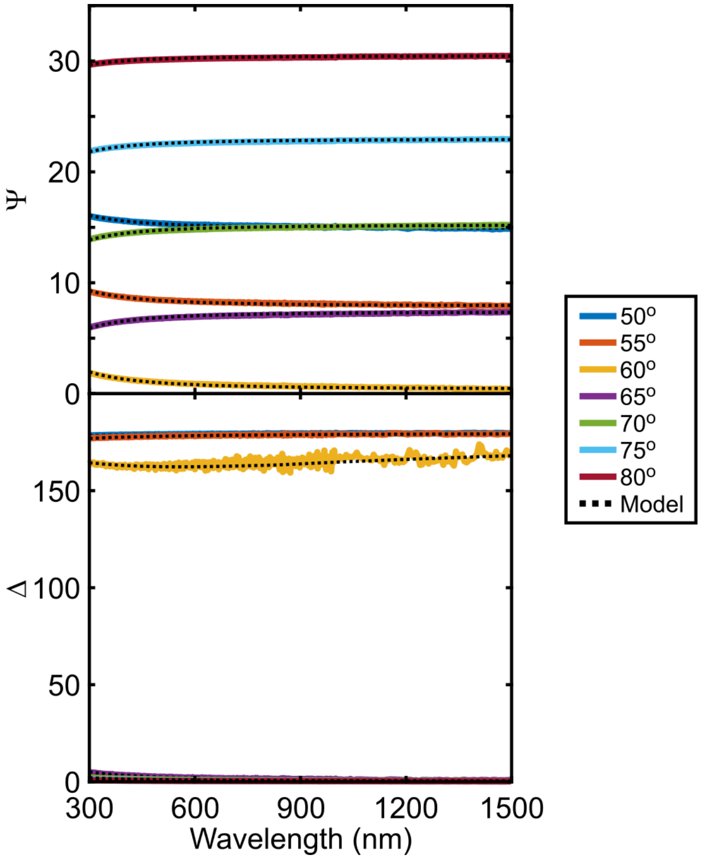

Supplementary Figure 2. Optical dispersion analysis of the Psi and Delta responses for single-side polished C-plane sapphire from standard reflectance spectroscopic ellipsometry.

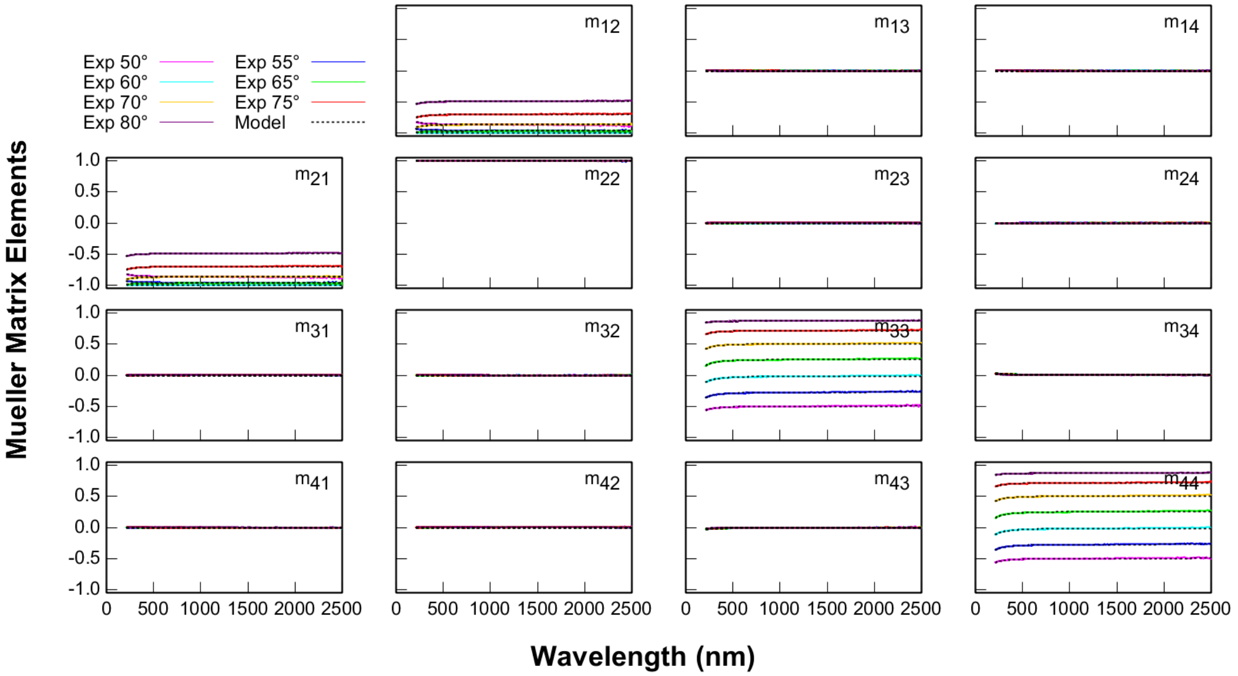

Supplementary Figure 3. Reflectance Mueller matrix spectroscopic ellipsometry data of single-side polished C-plane sapphire. All elements shown are normalized to the m_{11} element. Experimental angles of incidence are listed in the legend.

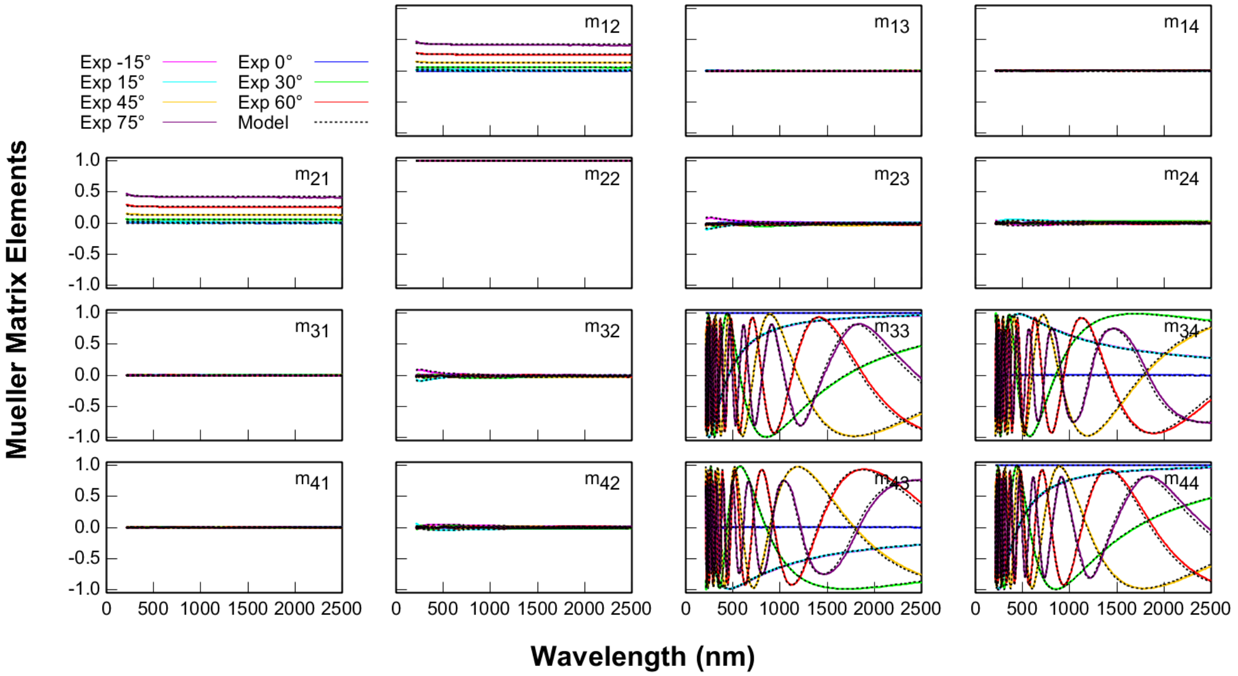

Supplementary Figure 4. Transmission Mueller matrix spectroscopic ellipsometry data of double-side polished C-plane sapphire. All elements shown are normalized to the m_{11} element. Experimental angles of incidence are listed in the legend.

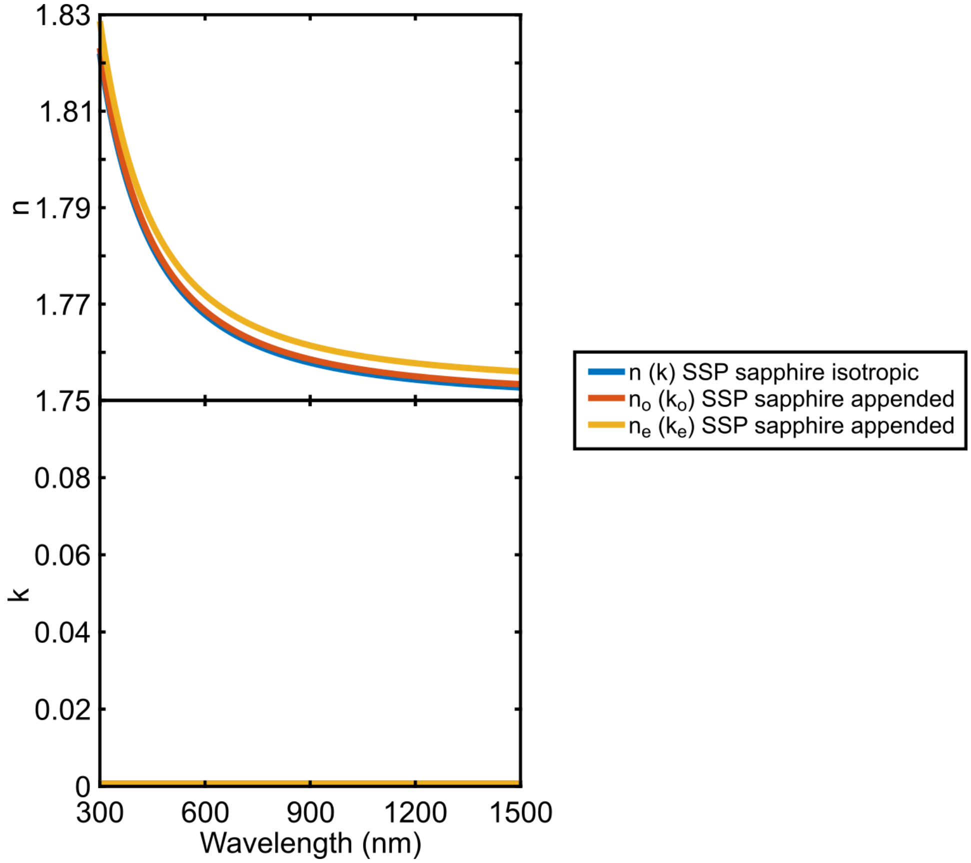

Supplementary Figure 5. The optical constants of C-plane sapphire used in this work. The isotropic response (n and k) is from standard ellipsometry and the appended response is from reflectance plus transmission Mueller matrix ellipsometry. The latter permits uniaxial anisotropic parameterization in the optical dispersion data analysis yielding the ordinary (n_o and k_o) and extraordinary (n_e and k_e) responses.

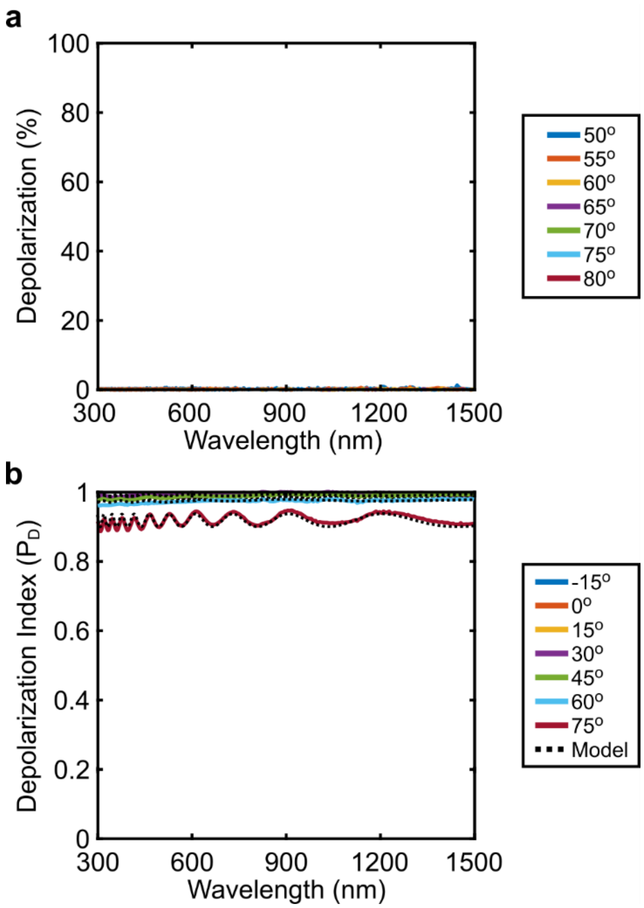

Supplementary Figure 6. Percent depolarization and the polarization index of the C-plane sapphire substrate. (**a**) Percent depolarization from reflectance spectroscopic ellipsometry of single-side polished C-plane sapphire. (**b**) P_D from transmission Mueller matrix ellipsometry of double-side polished C-plane sapphire.

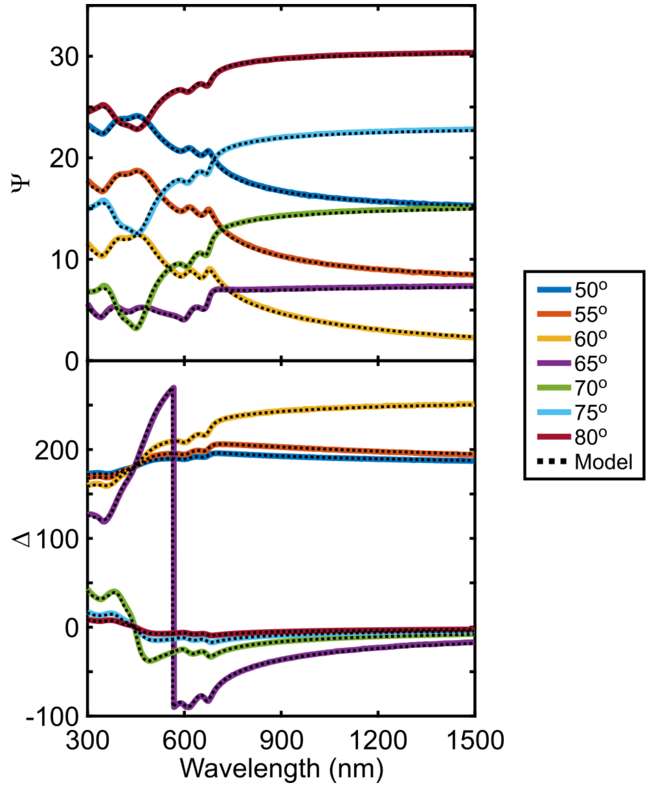

Supplementary Figure 7. Optical dispersion analysis of the Psi and Delta responses for NRE MoS_2 on single-side polished C-plane sapphire from standard reflectance spectroscopic ellipsometry.

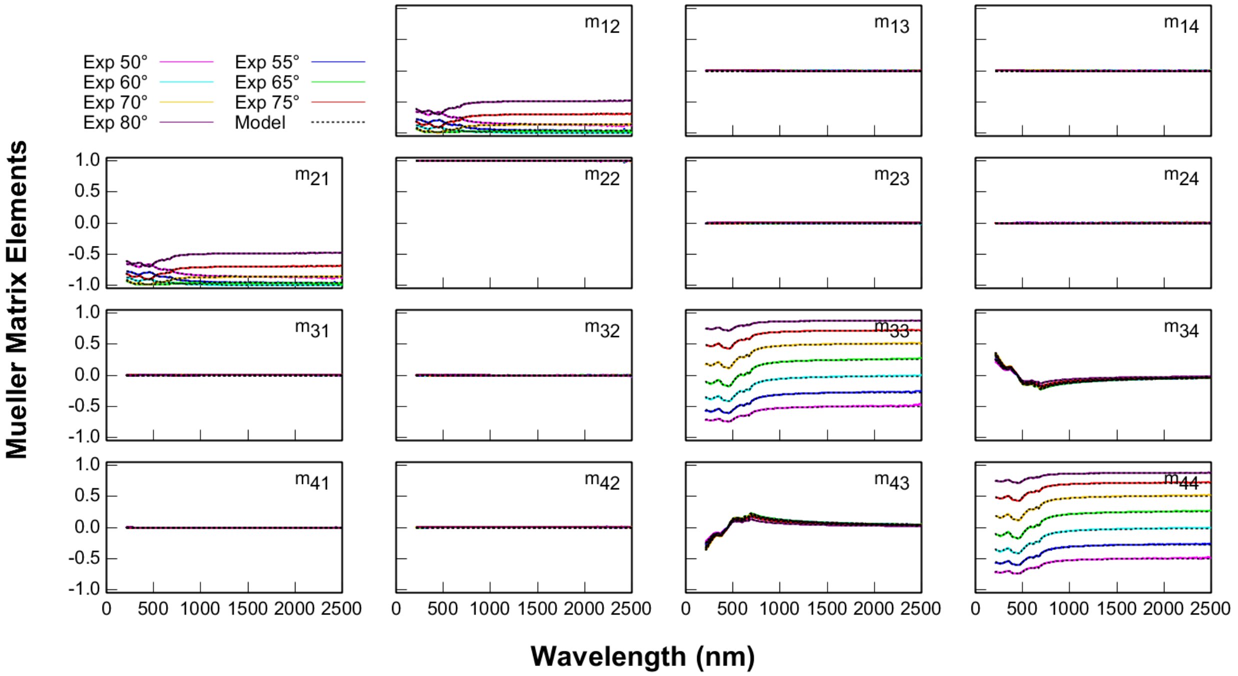

Supplementary Figure 8. Reflectance Mueller matrix spectroscopic ellipsometry data of NRE MoS_2 on single-side polished C-plane sapphire. All elements shown are normalized to the m_{11} element. Experimental angles of incidence are listed in the legend.

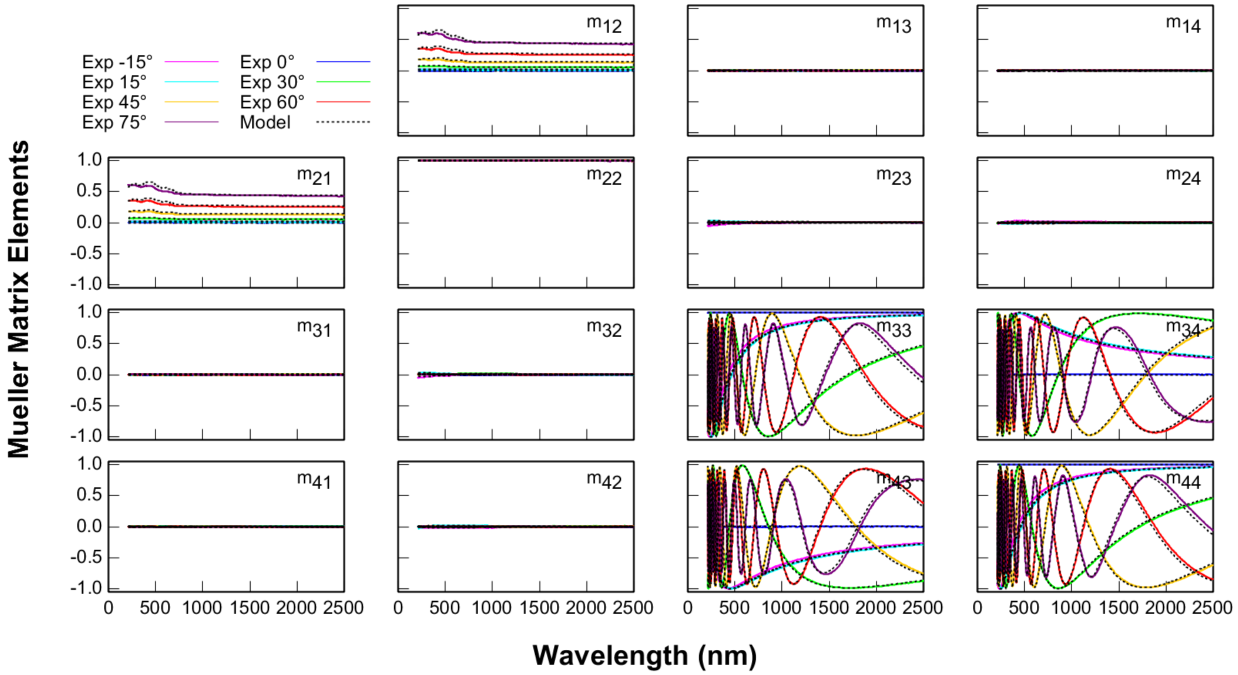

Supplementary Figure 9. Transmission Mueller matrix spectroscopic ellipsometry data of NRE MoS_2 on double-side polished C-plane sapphire. All elements shown are normalized to the m_{11} element. Experimental angles of incidence are listed in the legend.

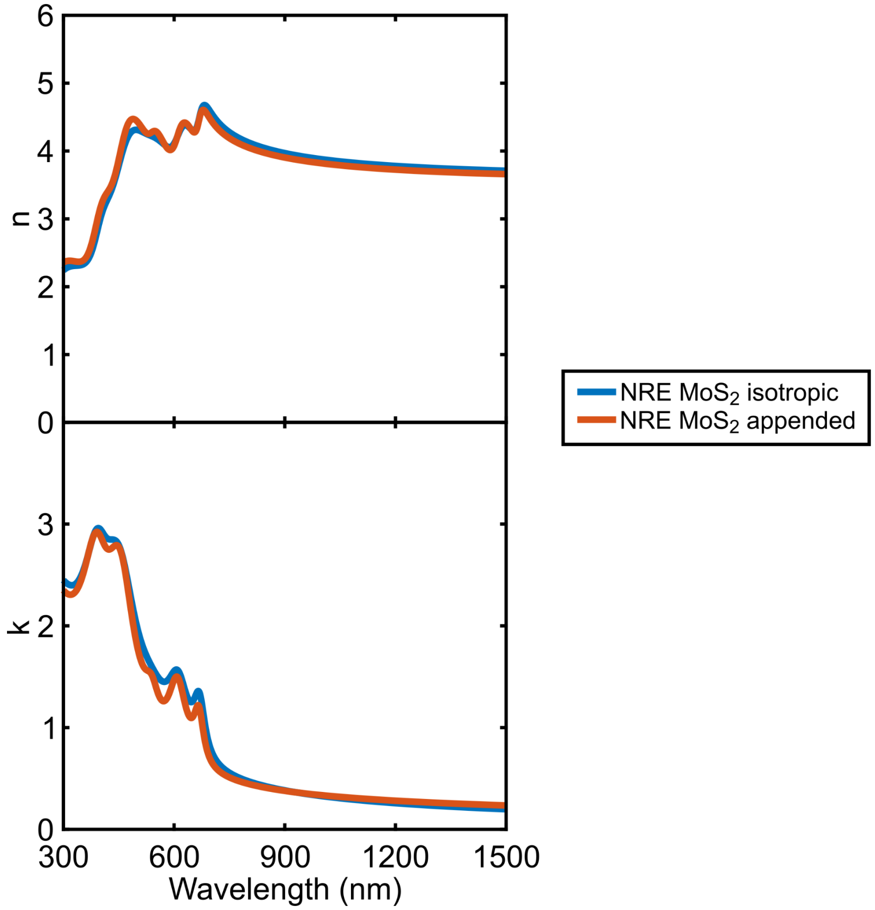

Supplementary Figure 10. The optical constants of NRE MoS_2 on C-plane sapphire. The isotropic response is from standard ellipsometry and the appended response is from reflectance + transmission Mueller matrix ellipsometry. An anisotropic model was not needed for the NRE MoS_2 film and is not used in the optical dispersion data analysis.

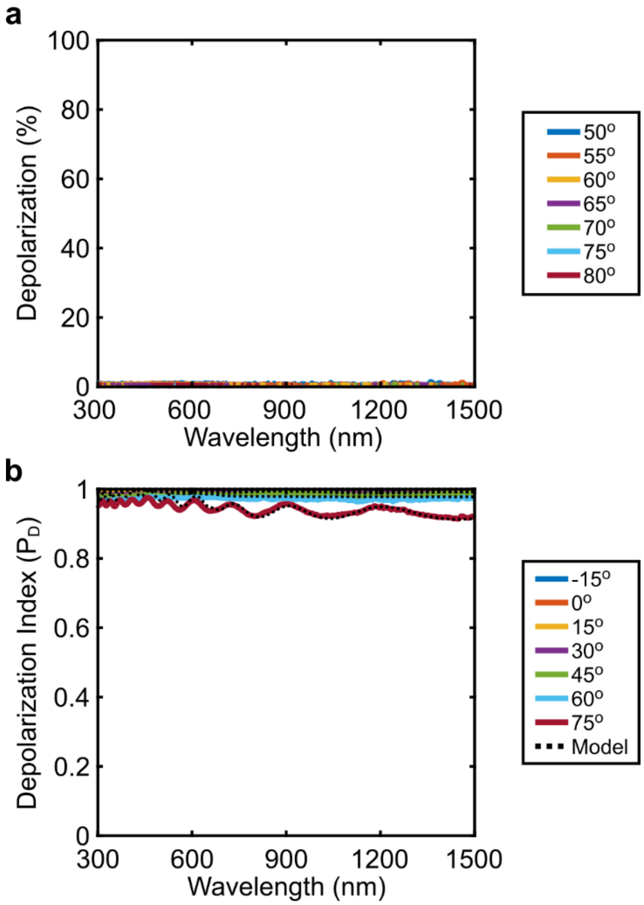

Supplementary Figure 11. Percent depolarization and the polarization index of thin film NRE MoS_2. (**a**) Percent depolarization from reflectance spectroscopic ellipsometry of NRE MoS_2 on single-side polished C-plane sapphire. (**b**) P_D from transmission Mueller matrix ellipsometry of NRE MoS_2 on double-side polished C-plane sapphire.

Supplementary Note 4. Modeled Quarter-Wave Optical Stacks

Illustrative optical material stacks involving exfoliated MoS_2 as the high-index material and PMMA as the low-index material were modeled using OptiLayer v.15.12. Here, we illustrate the modeled responses of representative quarter-wave optical stacks, or Bragg reflectors. A Bragg reflector is based on the half-wavelength spacing of high- and low-index layer materials[4,5]. This results in the symmetric periods of alternating high- and low-index materials and, based on the reference wavelength, can be tailored as a function of wavelength due to respective layer thicknesses. Throughout the design process, the reference wavelength of 1550 nm (vertical dashed line in Supplementary Figure 12 and 13) was selected for this design illustration, which is a common wavelength in optical telecommunication devices. This means the design iterations were adjusted with respect to the quarter-wave optical thickness for each material at this selected reference wavelength. The quarter-wave optical thickness (T_L) is defined as $T_L = 4N_L t_L cos\,\theta_L$ where N_L is the layer complex refractive index, t_L is the physical layer thickness, and θ_L is the incident angle for the respective material layer (L). Another simple way to design such optical stacks is to determine the physical thickness of the high- and low-index material layers using $t_H = \frac{\lambda}{4n_H}$ and $t_L = \frac{\lambda}{4n_L}$, respectively. This, however, does not always accurately incorporate influence from complex index materials (e.g., semiconductors or metals) where the extinction coefficient cannot be ignored at all wavelengths. Stack design parameterization details are provided in Supplementary Table 2. For our designs, actual n and k optical dispersion values from Fig. 2 were included in the respective models to generate the responses observed in Supplementary Figure 12 at 0° angle of incidence. Responses for off-angle incidence (45°) are shown in Supplementary Figure 13. PMMA was used as the low-index layer (optical dispersion data reported by Zhang et al.[6]). As an alternative, SME MoS_2 was used as the low-index material in the NRE MoS_2 / SME

MoS_2 design. Given the low refractive index response for SME MoS_2 (or RE MoS_2), this can also be used in optical coating design engineering as shown in Supplementary Figure 12f and 13e.

Supplementary Table 2. Quarter-wave optical stack characteristics for the model responses provided in Fig. 3 and Supplementary Figure 12 and 13.

Coating Characteristics	SME MoS_2 / PMMA	CEPR MoS_2 / PMMA	RE MoS_2 / PMMA	NRE MoS_2 / PMMA	NRE MoS_2 / SME MoS_2
Δn at 1550 nm	0.24	1.45	0.55	2.31	2.08
Δk at 1550 nm	0.07	0.24	0.12	0.15	0.07
Reflectance (%) at 1550 nm[a]	34	79	60	93	89
Avg. Reflectance (%)[b]	15	60	32	79	73
t_H (nm)	227	132	191	102	102
t_L (nm)	263	263	263	263	227
Number of Layers	7	7	7	7	7
Total Stack Thickness (nm)	1695	1318	1554	1198	1090

[a]Reflectance at AOI = 0°. [b]Reflectance is averaged over the wavelength range 1000-2000 nm.

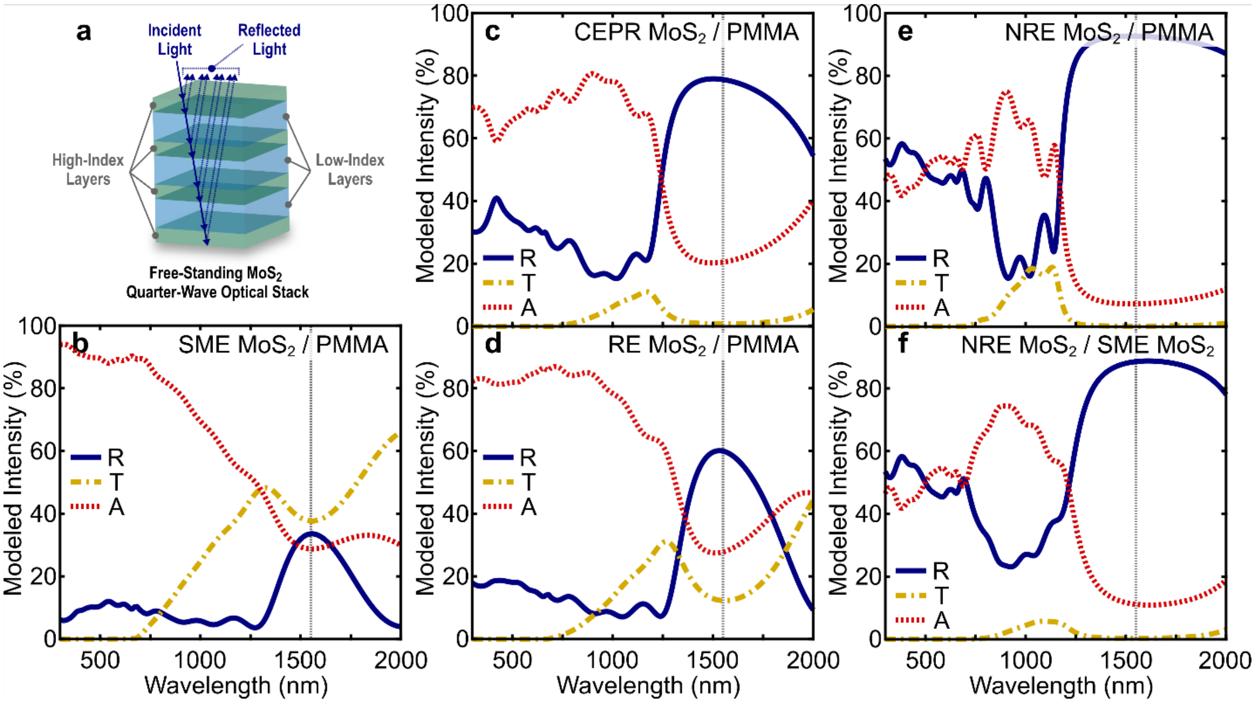

Supplementary Figure 12. Illustrative quarter-wave optical stacks. (**a**) Alternating high- and low-index material layer coatings. The modeled percent intensity (i.e., reflectance R, transmission T, and absorptance A) for these layer-by-layer optical stacks at normal (or 0°) angle of incidence are shown in for (**b**) SME MoS_2 / PMMA, (**c**) CEPR MoS_2 / PMMA, (**d**) RE MoS_2 / PMMA, (**e**) NRE MoS_2 / PMMA, and (**f**) NRE MoS_2 / SME MoS_2. The reference wavelength of 1550 nm (vertical dashed line) was selected for this design illustration, which is a common wavelength in optical telecommunication devices. The legend indicates the high-index layer (left) and the low-index layer (right). Most of the difference observed in the modeled reflectance is due to the index contrast (Δn) between the high- and low-index layers (see Supplementary Table 2).

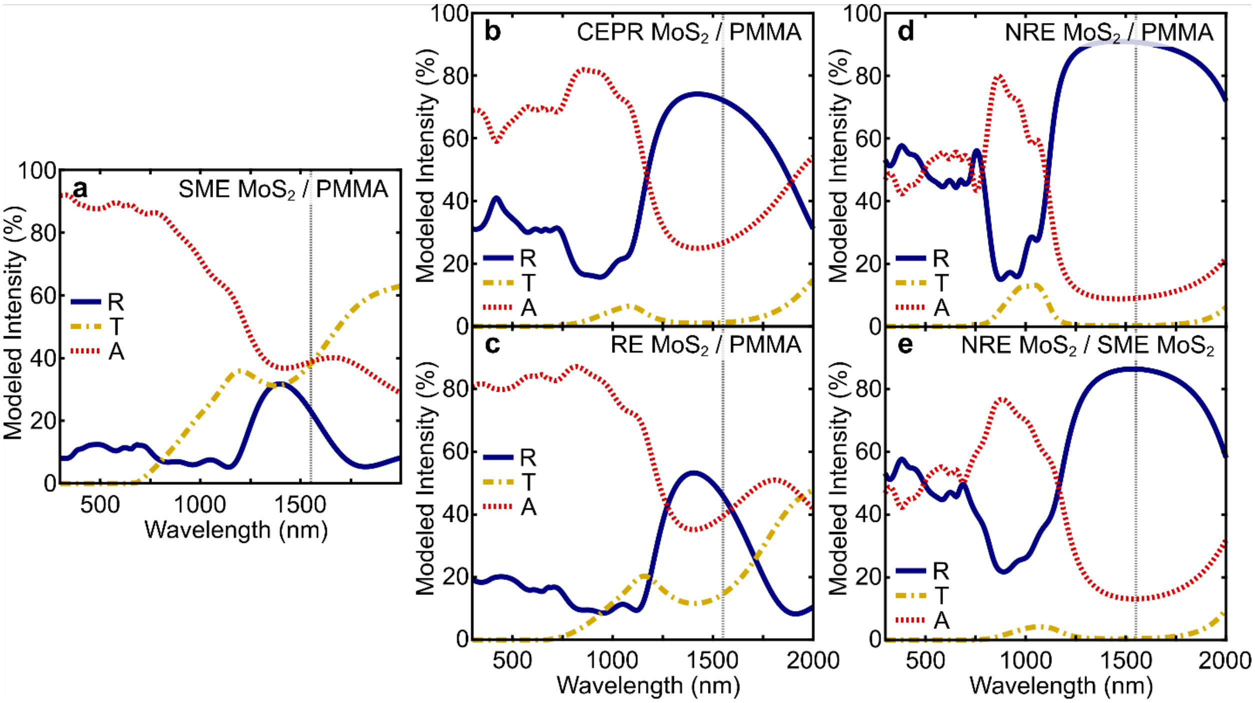

Supplementary Figure 13. The modeled percent intensity (i.e., reflectance R, transmission T, and absorptance A) for these layer-by-layer optical stacks at 45° angle of incidence. (**a**) SME MoS_2 / PMMA, (**b**) CEPR MoS_2 / PMMA, (**c**) RE MoS_2 / PMMA, (**d**) NRE MoS_2 / PMMA, and (**e**) NRE MoS_2 / SME MoS_2. The reference wavelength of 1550 nm (vertical dashed line) was selected for this design illustration, which is a common wavelength in optical telecommunication devices. The legend indicates the high-index layer (left) and the low-index layer (right). Most of the difference observed in the modeled reflectance is due to the index contrast (Δn) between the high- and low-index layers (see Supplementary Table 2).

Supplementary Note 5. XPS Analysis

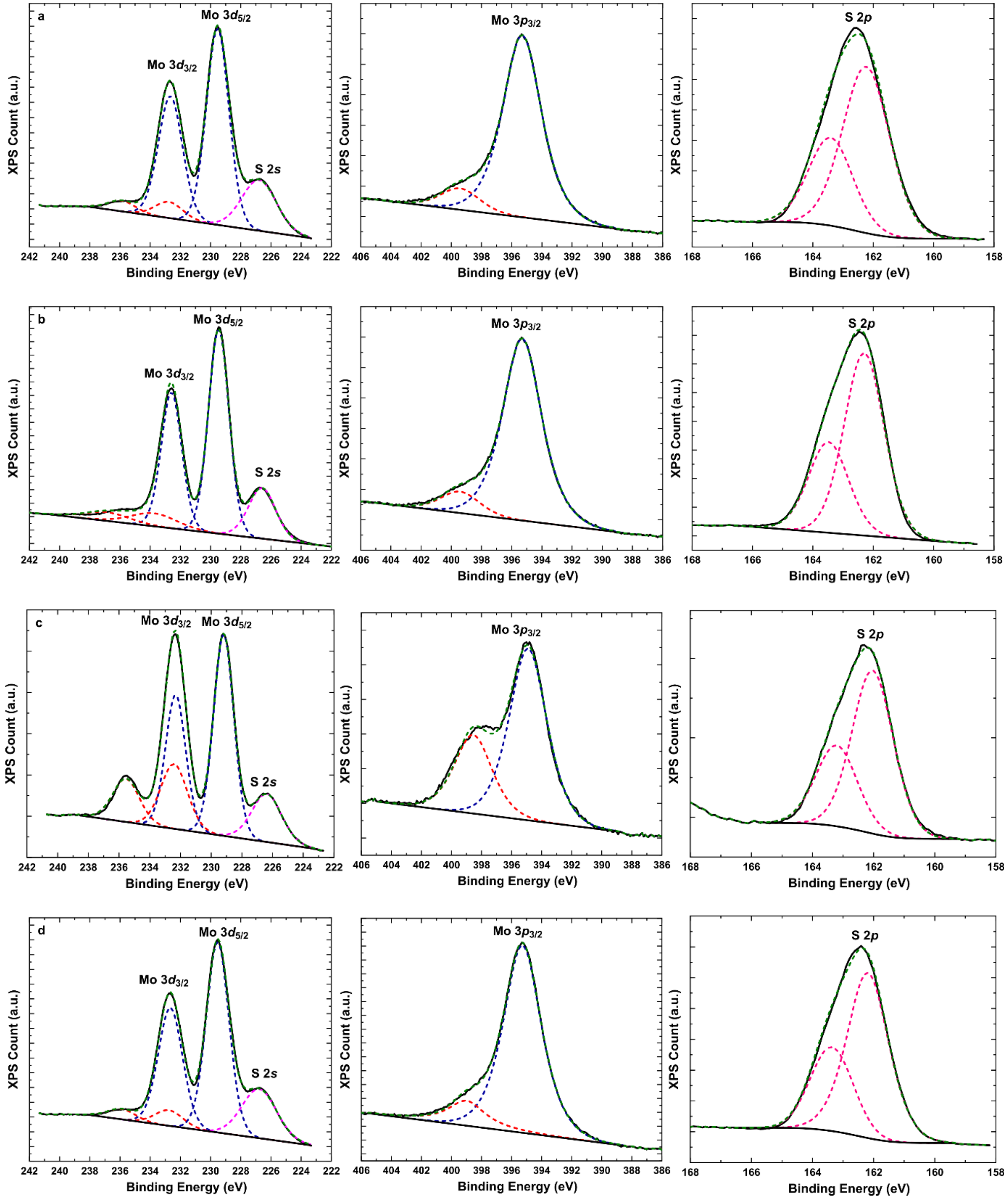

Supplementary Figure 14. XPS fits for exfoliated MoS_2 thin films. Mo 3*d* and S 2*s*, Mo 3*p*, and S 2*p* spectra of (**a**) SME MoS_2, (**b**) CEPR MoS_2, (**c**) RE MoS_2, and (**d**) NRE MoS_2 films. The Mo^{4+} (blue dash) at 229.4 eV and 232.5 eV for Mo 3$d_{5/2}$ and Mo 3$d_{3/2}$, 395.2 eV for Mo 3$p_{3/2}$. The Mo^{6+} (red dash) at 233.0 eV and 236.2 eV for Mo 3$d_{5/2}$ and Mo 3$d_{3/2}$, at 399.0 eV for Mo 3$p_{3/2}$.

Supplementary Table 3. Mo^{4+} (%) and Mo^{6+} (%) from the Mo $3p_{3/2}$, O/Mo, S/Mo, and S/Mo^{4+} ratios.

	Mo $3p_{3/2}$					
MoS_2	Mo^{4+} (%)	Mo^{6+} (%)	O/Mo	S/Mo	S/Mo^{4+}	f[a]
SME	90.0	10.0	0.51	2.35	2.61	0.10
PR	82.1	17.9	0.66	2.23	2.72	0.18
ARE	68.6	31.4	6.23	1.84	2.69	0.31
NRE	86.7	13.3	0.41	2.25	2.60	0.13
Sigma[b]	94.2	5.8	0.77	2.49	2.65	0.06
CVT[c]	97.1	2.9	0.72	2.54	2.61	0.03

[a]The percent oxidation $f = \frac{A(MoO_x)}{A(MoO_x)+A(MoS_2)}$ for the exfoliated MoS_2. [b]Sigma-Aldrich commercial source powder. [c]CVT source powder.

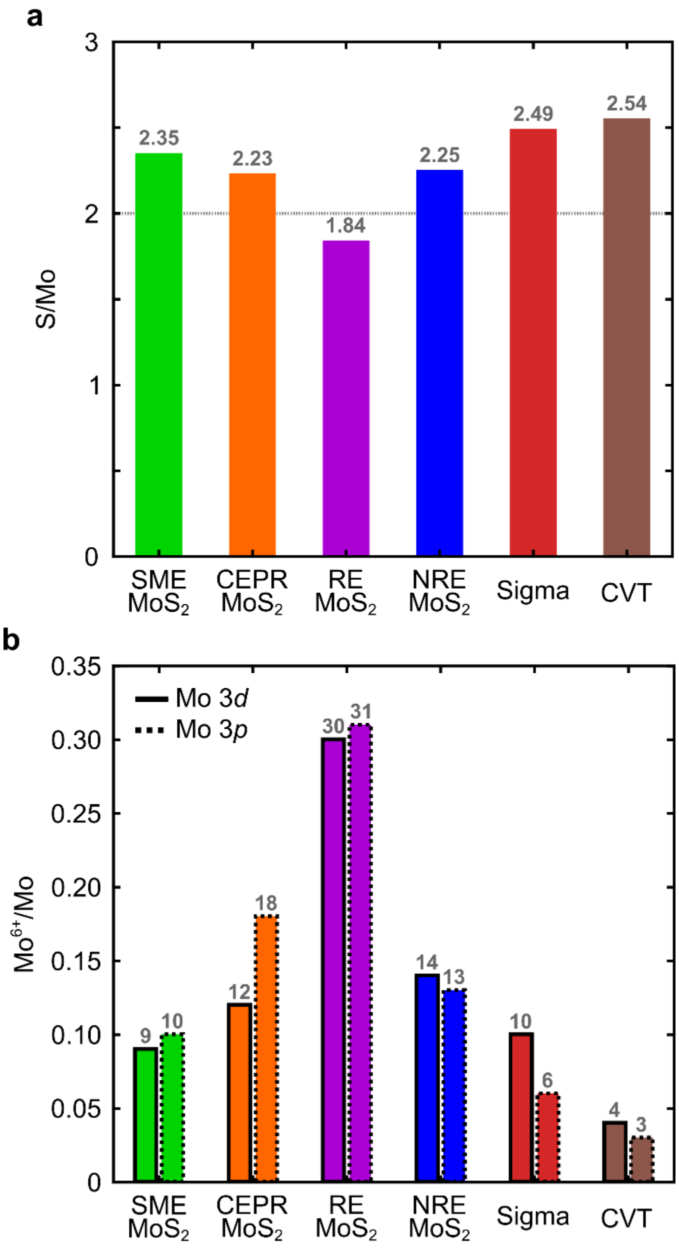

Supplementary Figure 15. XPS analysis of all exfoliated MoS_2 and starting source powders. (**a**) The S/Mo ratios from the Mo 3*p* and S 2*p* fitted peaks and (**b**) the oxidation fractions from Mo 3*d* and S 2*s* fitted peaks (solid outline) and from the Mo 3*p* and S 2*p* fitted peaks (dashed outline). Note that all the exfoliated MoS_2 was prepared using the CVT MoS_2 source powder. Untreated commercial Sigma (Sigma-Aldrich) source powder is shown here for comparison to our untreated CVT MoS_2 source powder.

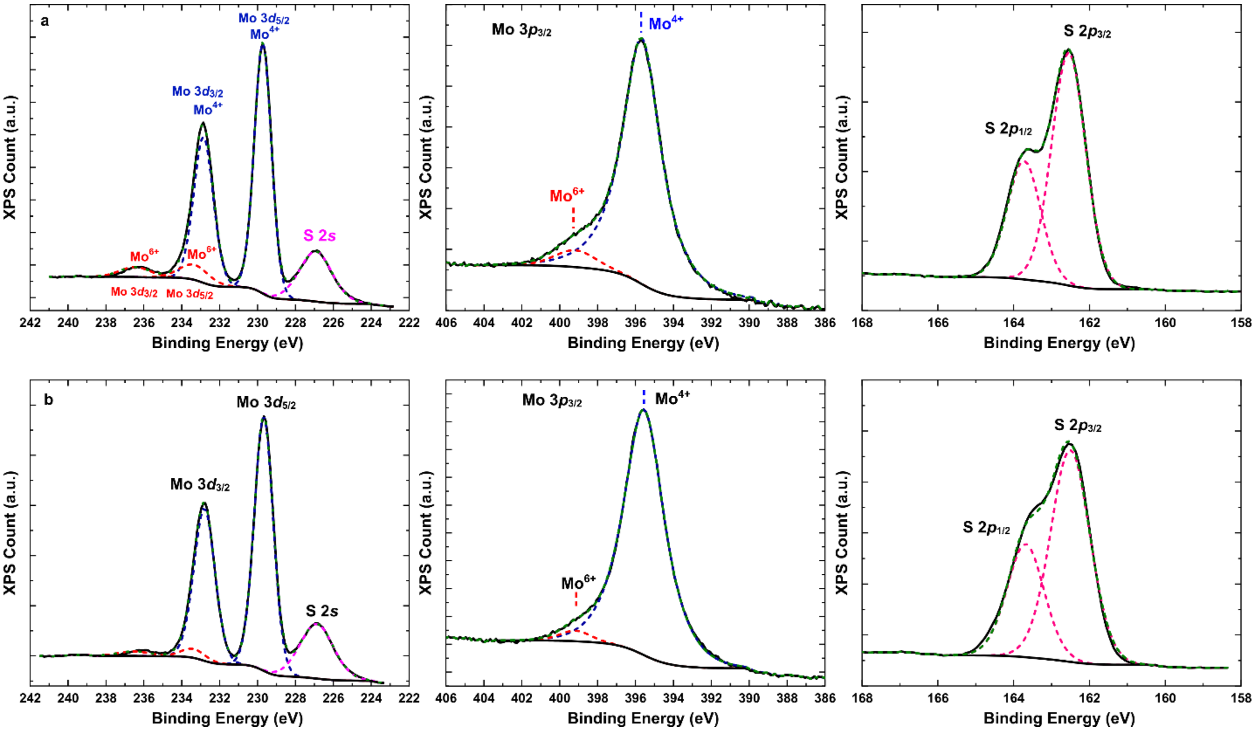

Supplementary Figure 16. XPS fits for the MoS_2 commercial (Sigma) and CVT source powders. Mo 3*d*, Mo $3p_{3/2}$ and S 2*p* spectra of (**a**) Sigma MoS_2 and (**b**) CVT MoS_2. The Mo^{4+} (blue dash) at 229.7 eV and 232.8 eV for Mo $3d_{5/2}$ and Mo $3d_{3/2}$, 395.7 eV for Mo $3p_{3/2}$. The Mo^{6+} (red dash) at 233.3 eV and 236.5 eV for Mo $3d_{5/2}$ and Mo $3d_{3/2}$, at 399.2 eV for Mo $3p_{3/2}$.

Supplementary Note 6. Additional Raman Analysis

Resonant Raman spectra were acquired for each powder source: Sigma-Aldrich and CVT MoS_2 for comparison. The resulting spectra and their fits are shown in Supplementary Figure 17 below. These fits included peaks around 180, 185, 210, and 225 cm^{-1}, the last of which corresponds to the LA mode. As in the case of the exfoliated MoS_2 samples, the LA/E_{2g} peak intensity ratio was extracted. The results for each film type and powder source are shown in Supplementary Figure 18.

A small peak near 750 cm^{-1} is present for each MoS_2 film in the 514.5 nm Raman spectra (Fig. 6b), consistent with the B_{2g} peak of MoO_2. In order to account for variations in the intensity of this peak due to differences in sample thickness and crystallinity, each spectra was normalized with respect to the intensity of the MoS_2 A_{1g} peak. While the resulting peak intensities are not true representations of MoO_2 concentration, they do suggest that a greater amount of oxidation in NRE MoS_2 is in the form of MoO_2 when compared to the other MoS_2 films (Supplementary Figure 19).

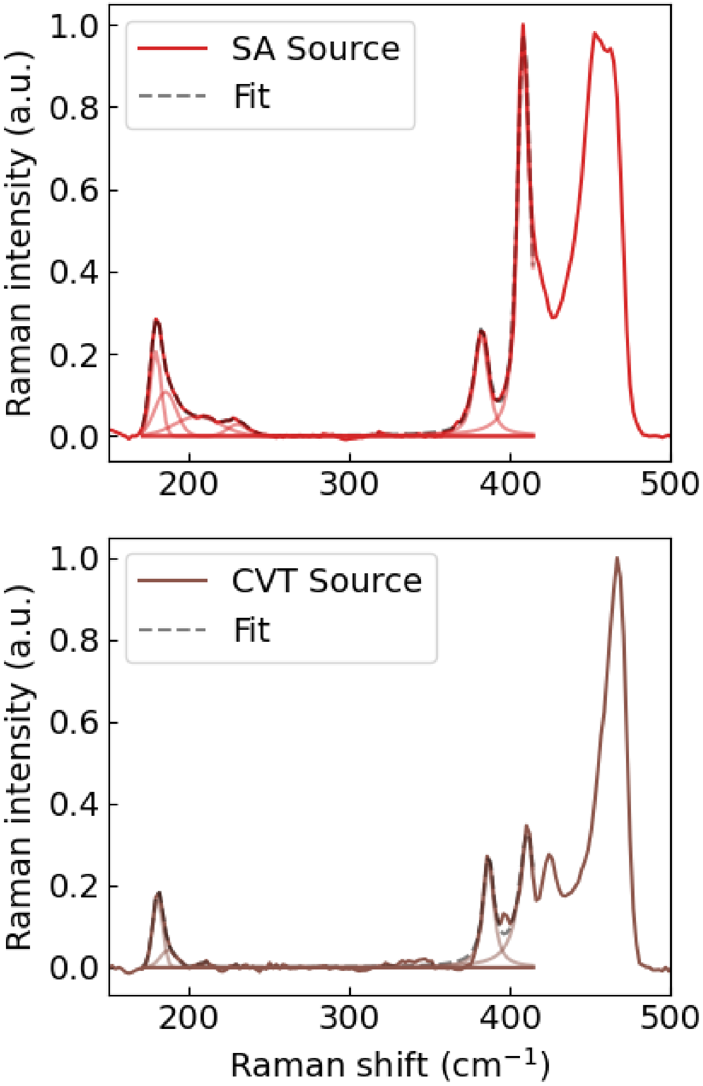

Supplementary Figure 17. 633 nm Raman spectra for each of the source Sigma-Aldrich (SA) and CVT powders. The corresponding fits are also shown, which included the LA, E_{2g}, and A_{1g} peaks, as well as additional peaks at 180, 185, and 210 cm^{-1}.

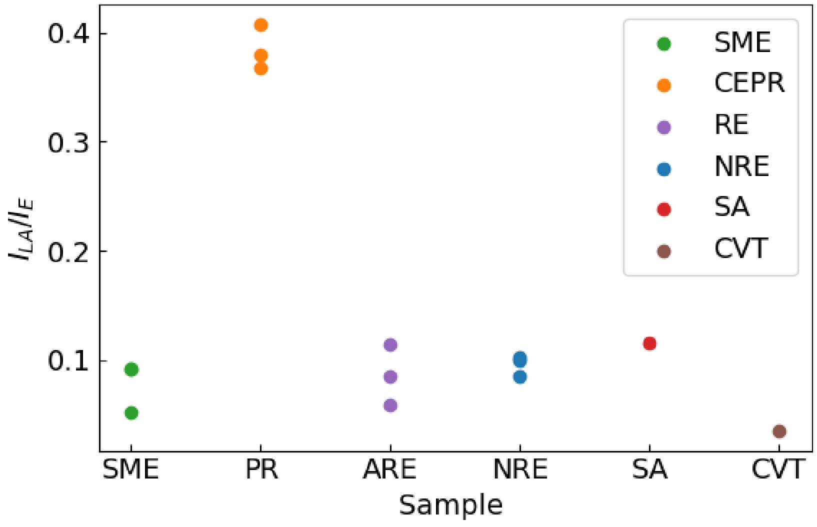

Supplementary Figure 18. Summary of the LA/E_{2g} intensity ratios for each exfoliated MoS_2 type as well as the two powder sources for comparison.

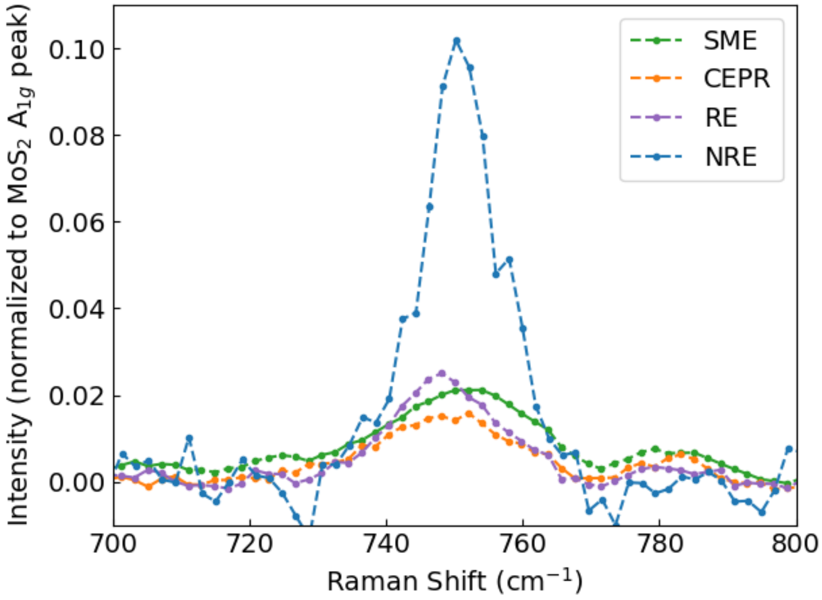

Supplementary Figure 19. 514.5 nm Raman spectra of each MoS_2 type, centered around a peak near 750 cm^{-1} that is consistent with the B_{2g} peak of MoO_2. The intensity of the spectra have been normalized to the intensity of the A_{1g} peak of MoS_2.

Supplementary Note 7. Impact of POMs on the Optical Properties of Monolayer MOCVD MoS_2 Films

The RE MoS_2 film optical properties necessitate consideration of known complexes that carry out the exfoliation process. As discussed in the main text, POM species represent oxidation pathways artificially utilized to facilitate TMDC exfoliation. Unfortunately, characterization of POMs is challenging due to their complex morphologies and environmentally-dependent equilibria present in solution. The native metal oxide formation on surfaces and edges of TMDCs further poses a compositional complexity. Additionally, the inability to readily remove POMs in solution from RE MoS_2 complicates a direct correlation as to what impact these exfoliation species have on the optical response for liquid phase exfoliated MoS_2. As an alternative approach, a controlled study is presented in Supplementary Figure 20a showing metalorganic chemical vapor deposited (MOCVD) MoS_2 films without, with, and rinsed of molybdenum (Mo) POMs used in the exfoliation of RE MoS_2. Mo POMs were prepared and isolated by retaining MOPs from oxidizing the bulk powder source and centrifuging the liquid to remove any remaining bulk powder MoS_2 (i.e., non-exfoliated layered MoS_2 in the solution). The isolated MOPs were then treated with the same reducing agent discussed in the main text resulting in a visually blue solution upon POM formation. This solution is considered an analogue for the POMs formed in situ during the artificial redox exfoliation process (as they represent the same chemistry but without the presence of bulk MoS_2 powders leading to subsequent MoS_2 exfoliation).

Baseline optical properties for an as-prepared MOCVD MoS_2 film are shown in Supplementary Figure 20b,c. This same MOCVD MoS_2 film was then dip coated in a solution of Mo POMs in acetonitrile (ACN, approximately 0.005 mg/ml) and dried under N_2. The optical

properties for this MOCVD MoS_2 film with Mo POMs is shown in Supplementary Figure 20b,c with a decrease in the complex refractive index. This MOCVD MoS_2 film with Mo POMs was then rinsed to assess how reversible the response is after attempting to remove the POM adsorbates. ACN was initially used to remove the dip coated Mo POMs (the same solution used for RE MoS_2 suspensions and isolated Mo POMs), but no change in the optical response was observed. As a result, a more aggressive solvent rinse was used involving a 3-5 minute soak in dimethylformamide (DMF) and then tetrahydrofuran (THF). This solvent rinse results in a partial regeneration of optical properties observed in Supplementary Figure 20b,c. Unexpectedly, solvent rinsing does not produce full reversibility in the optical response as has been shown with organic adsorbates in similar work[7]. This observation suggests Mo POM complexes remain on the surface/edges after rinsing and/or the more aggressive solvent wash degrades the MOCVD MoS_2 film. To isolate the influence of the Mo POM solvent rinse, AFM images for the as-prepared MOCVD MoS_2 film (Supplementary Figure 20d), MOCVD MoS_2 film with Mo POMs (Supplementary Figure 20e), and MOCVD MoS_2 rinsed film (Supplementary Figure 20f) show residual surface adsorbates. As a result, we attribute the change in optical response in Supplementary Figure 20b,c primarily due to Mo POMs on the MOCVD MoS_2 film surface.

The modulation of optical properties observed here resembles dopant-induced screening described in previous work[7]. As expected, due to the negative charge of the POMs, a decrease in n and a redshifting of exciton peaks is observed indicative of an n-type dopant effect[8]. Characteristic of dopant-induced screening, this decrease in n is likewise observed at higher energies resulting in broadband changes in the optical properties. This is further observed in the $\Delta(n,k)/(n,k)$ plots in Supplementary Figure 20b,c. Here, $\Delta(n,k)/(n,k)$ describes the difference between as-prepared MOCVD MoS_2 and the respective Mo POM film condition (i.e., after dip

coating and after rinsing with DMF and THF). The $\Delta(n,k)/(n,k)$ characteristics for the Mo POM film conditions are similar, suggesting comparable influences for each film. While POMs represent an important contribution to the variability observed in Fig. 2, the MOCVD MoS_2 $\Delta(n,k)/(n,k)$ data in Supplementary Figure 20b,c suggest additional influence to the optical response (i.e., sulfur vacancies and high oxidation from XPS data in Fig. 5). We also note that the effects illustrated in Supplementary Figure 20 assumes ex situ POM formation mimics in situ exfoliation kinetics, in relation to both POM composition and interfacial surface chemistry, which is unlikely.

Metalorganic chemical vapor deposition of monolayer MoS_2 was carried out on C-plane sapphire substrates placed in the hot-zone of a horizontal quartz tube reactor. H_2 was used as the carrier gas to deliver volatilized $Mo(CO)_6$ (99.9%, Sigma-Aldrich) to the furnace during film growth. Sulfur was supplied by flowing high purity H_2S (99.5%, Sigma-Aldrich) into the chamber. Synthesis was performed in a chalcogen rich environment with a S/Mo ratio of $\sim 10^4$. Argon gas was used as the push gas to drive gaseous precursors down the tube and ensure a steady flux of $Mo(CO)_6$ and H_2S to the substrate during growth. Film growth was carried out at 900 °C with a reactor pressure of 50 Torr. A three-step growth process was employed to better control nucleation density and achieve uniform monolayer MoS_2 films[9].

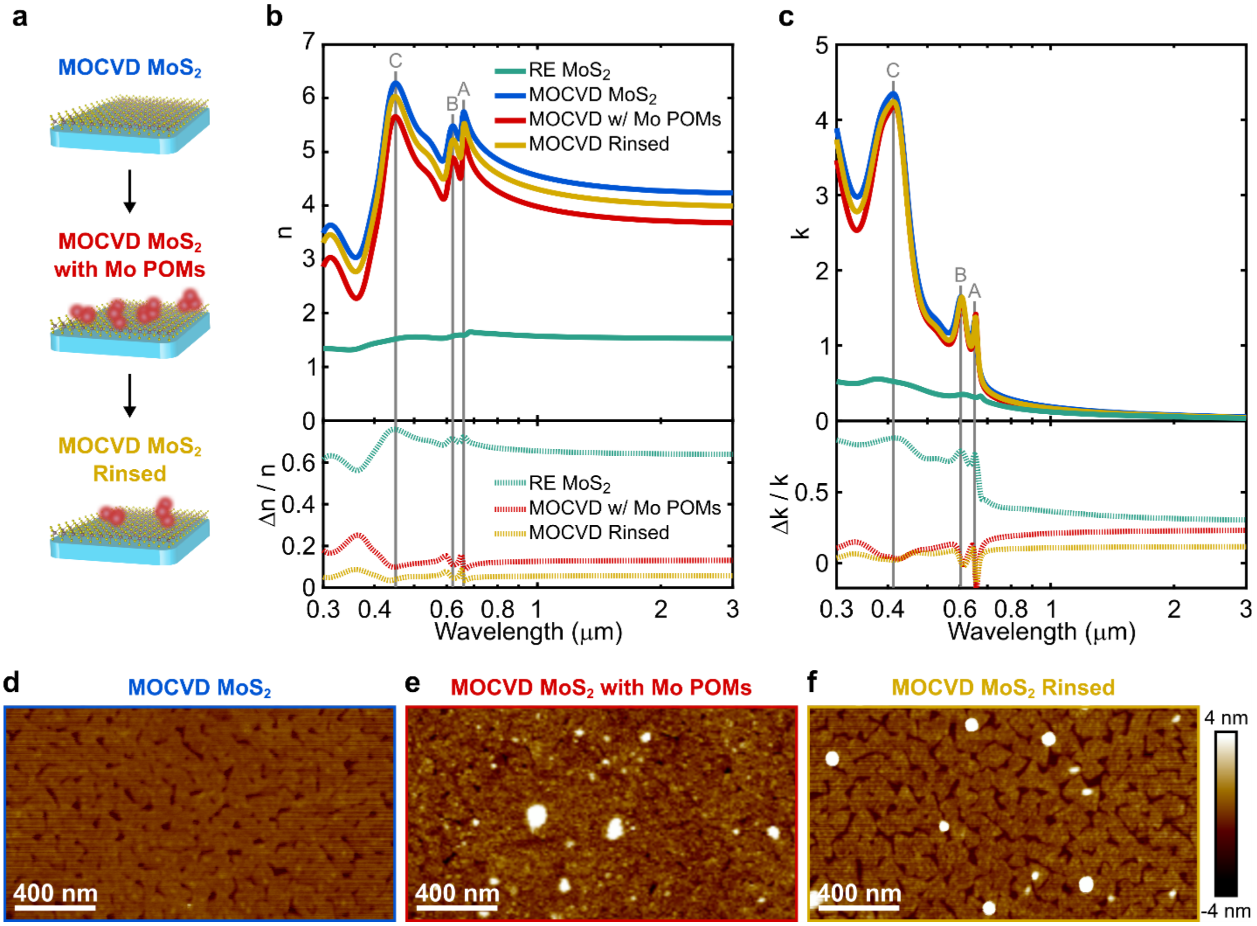

Supplementary Figure 20. Illustration of RE MoS_2 exfoliation species (i.e., Mo POMs) on the optical properties of as-prepared MOCVD MoS_2. (**a**) Schematic representation of the different Mo POM film conditions. (**b**) n and $\Delta n/n$ plots for the films in (**a**). **c** k and $\Delta k/k$ plots for films in (**a**). AFM micrographs of the films described in (**a**) including the (**d**) as-prepared MOCVD MoS_2 film, (**e**) MOCVD MoS_2 film with Mo POMs, and (**f**) rinsed MOCVD MoS_2 film after treating with Mo POMs.

Supplementary Note 8. Computational Details

All calculations were carried out using the Vienna ab initio simulation package (VASP)[10,11]. The Kohn-Sham equations were solved using a plane wave basis set with an energy cutoff of 500 eV, and the projector augmented-wave (PAW) potential was applied. Structures were optimized with the Perdew−Burke−Ernzerhof (PBE) exchange-correlation functional[12]. Structural optimization was converged to the force within 0.01 eV/Å. Electronic structure calculations were performed using PBE, unless indicated otherwise, and spin-orbit coupling (SOC) was included. Optical absorption spectra were calculated at the G_0W_0-BSE level based on DFT/PBE, and including SOC. The imaginary part of the dielectric function $\varepsilon^{(2)}$ (where $\varepsilon = \varepsilon^{(1)} + i\varepsilon^{(2)}$) is given by the Cartesian tensor[13],

$$\varepsilon_{\alpha\beta}^{(2)}(\omega) = \frac{4\pi^2 e^2}{\Omega} \lim_{q\to 0} \frac{1}{q^2} \sum_{c,v,\vec{k}} 2\omega_{\vec{k}} \delta(\varepsilon_{c\vec{k}} - \varepsilon_{cv} - \omega) \times \left\langle u_{c\vec{k}+\vec{e}_\alpha q} \middle| u_{v\vec{k}} \right\rangle \left\langle u_{c\vec{k}+\vec{e}_\beta q} \middle| u_{v\vec{k}} \right\rangle^*, \qquad (3)$$

where ω is in units of energy; the volume of the cell; $\vec{e}$ unit vectors for the three Cartesian directions; c and v refer to conduction and valence states, respectively; $u_{c\vec{k}}$ are the cell-periodic orbitals at $\vec{k}$. In the calculation of $\varepsilon_{\alpha\beta}^{(2)}(\omega)$, $\vec{k}$ is restricted to the irreducible wedge of the first Brillouin zone. The real part of the dielectric tensor is obtained from the Kramers-Kronig transformation and given by

$$\varepsilon_{\alpha\beta}^{(1)}(\omega) = 1 + \frac{2}{\pi} P \int_0^\infty \frac{\varepsilon_{\alpha\beta}^{(2)}(\omega')\omega'}{\omega'^2 - \omega^2 + i\eta} d\omega', \qquad (4)$$

where P denotes the principal value of the integral. Spectra were calculated by

$$\alpha_{ij}(\omega) = \frac{\sqrt{2}}{ch} E \sqrt{\sqrt{\varepsilon_{ij}{}^{(1)}(\omega)^2 + \varepsilon_{ij}{}^{(2)}(\omega)^2} - \varepsilon_{ij}{}^{(1)}(\omega)}, \qquad (5)$$

where $i,j=x,y,z$. We used the average absorption coefficient, defined by

$$\alpha = \frac{\alpha_{xx}+\alpha_{yy}+\alpha_{zz}}{3}. \qquad (6)$$

Monolayer MoS_2 structures were modeled by a hexagonal 3×3×1supercell (9 Mo and 18 S atoms) with k sampling of 5×5×1. A 15 Å vacuum was added perpendicular to the MoS_2 monolayer plane. A larger supercell of 5×5×1 was also used with a k sampling of 3×3×1. The ENCUTGW parameter (energy cutoff for the response function) was 267 eV, and NBANDS (number of bands) was 300. Pristine monolayer MoS_2 and with a single S vacancy (SV) were considered (see Fig. 7a). Both lattice constants and atomic coordinates were optimized.

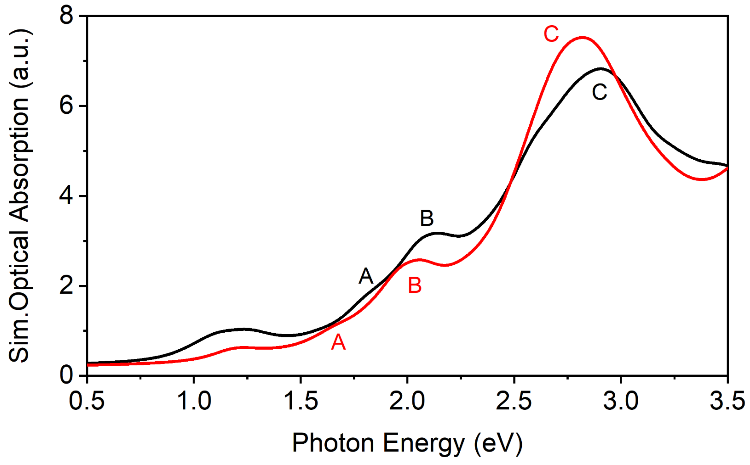

Supplementary Figure 21. PBE-SOC optical absorption spectra for MoS_2 with a SV for 3×3×1 (black line) and 5×5×1 (red line) supercells.

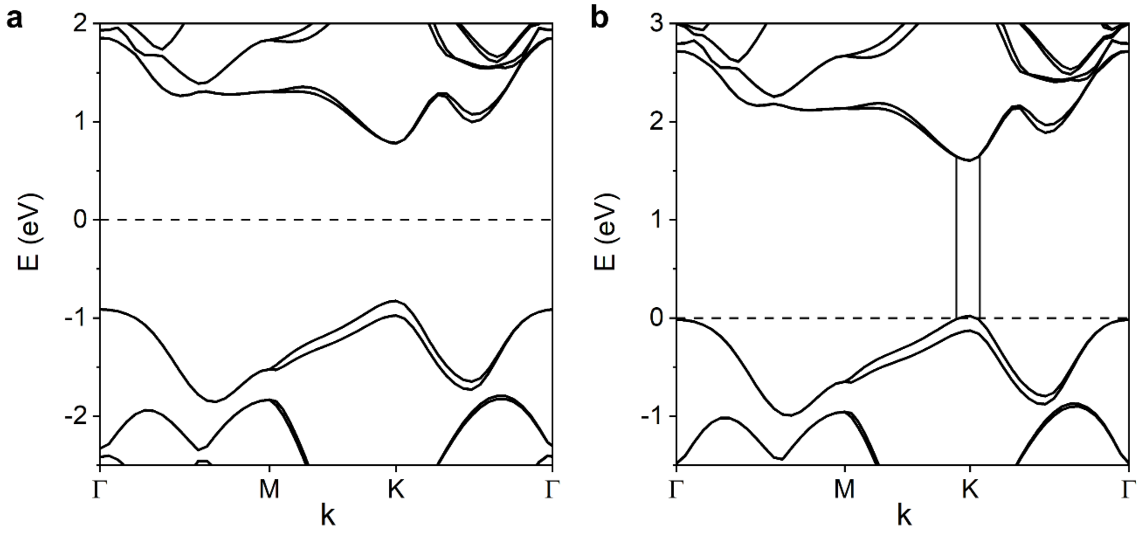

Supplementary Figure 22. PBE-SOC band structures. Monolayer (**a**) pristine MoS_2 and (**b**) MoS_2 with less 0.1e. The Fermi level is set at E = 0. In (**b**), optical transitions in the region between the denoted two vertical lines are forbidden.

Supplementary Table 4. The peak exciton energy positions in the optical absorption spectra for pristine MoS_2 and MoS_2 with a S vacancy compared to the exfoliated MoS_2 (Fig. 2e).

MoS_2	Defect	A Exciton (eV)	B Exciton (eV)	C Exciton (eV)
Theory				
Pristine	-	1.89	2.15	2.91
S Vacancy	1.40	2.07	2.25	3.24
Experiment				
NRE MoS_2	-	1.87	2.08	3.26
CEPR MoS_2	-	1.90	2.10	3.00
RE MoS_2	-	1.85	2.07	-
SME MoS_2	-	1.85	2.05	-

Supplementary Note 9. Starting Source Powder and Comparison

Supplementary Figure 23 shows a comparison of optical properties involving semiconducting exfoliated MoS_2 from the different exfoliation methods (see Fig. 1). In most cases, the observed n and k for each MoS_2 type is further suppressed due to the use of the commercial pretreated MoS_2 powder (Sigma-Aldrich, 15 μm powder, lot: WXBC8313V) in comparison to our in-house untreated chemical vapor transport (CVT) MoS_2 source powder. The pretreatment of the Sigma powder follows procedures outlined in prior work[14]. Note that in the case of CEPR MoS_2, the n increases with the use of the Sigma source powder. We suspect this is due to processing-dependent interactions primarily between the phase reconversion, doping characteristics, and lattice strain. This suggests that even further optical response tailorability is possible between phase reconfiguration and the material state (i.e., defect engineering via lattice vacancies and oxidation). Note that NRE MoS_2 optical properties drop significantly due to the use of the pretreated commercial starting source, further illustrating the sensitivity of the resulting optical properties. Indeed, NRE MoS_2 begins to resemble the optical properties of both SME and ARE MoS_2 (irrespective of starting source) due to the use of the pretreated commercial Sigma powder.

Scanning electron microscope (SEM) based chemical analysis was conducted using a Thermo Fisher Scientific Apero C FEG SEM with an Oxford Instruments X-Max 65 energy dispersive spectroscopy (EDS) detector. Measurements were taken with an accelerating voltage of 5 kV and a probe current of 3.2 nA. Source powder samples were prepared by placing the powder on double sided carbon tape affixed to a 10 mm diameter aluminum holder.

SEM micrographs illustrate significant differences in the starting bulk powder dimension. Such changes in surface area will influence the oxidation of such powders and the resulting chemistry of exfoliation employed. The EDS analysis confirms XPS stoichiometry of MoS_2 in the

CVT and Sigma powders. In addition to molybdenum and sulfur, low amounts of silicon and oxygen were detected in both samples (not observed in XPS of the powders), as shown in the spectra in Supplementary Figure 24 and 25. For CVT MoS_2, silicon and oxygen are concentrated in discrete particles (as outlined by dashed circles on the EDS maps in Supplementary Figure 25d,e), which appear to be adsorbed on the MoS_2 powder surface as shown in the secondary electron SEM image (Supplementary Figure 25a). This implies that these particles are likely surface contaminants introduced post growth. For CVT MoS_2, post-growth contamination may occur when the ampoule is broken to retrieve the bulk crystal MoS_2. For the Sigma powder, the source of the SiO_x contamination is unknown. However, in both cases, SiO_x contamination may come from the purity of the respective growth precursors. Due to the low concentration of these particles in the analysis, as conducted in this study, it was not possible to determine the stoichiometry of the SiO_x compound. Overall, the bulk composition measurements further suggest relatively pure MoS_2 powder, with the only impurities being the low amounts of SiO_x particles.

In Supplementary Figure 26, we show the XRD pattern comparing two sources of MoS_2, both in-house grown CVT powder and commercially available powder from Sigma. Powder patterns were taken using a Rigaku Smartlab system. The incident radiation was CuK_α (1.5418 Å). Lattice parameters for the in-house sample were *a,b*: 3.16354(12) Å and *c*: 12.29281(25) Å. In comparison, the Sigma powder evinced lattice parameters of *a,b*: 3.16097(5) Å and *c*: 12.30127(17) Å. For comparison, in previous publications[15], the values are listed as *a,b*: 3.163 Å and *c*: 12.247 Å when placed in a standardized setting. From the XRD graph, we observe in-house grown CVT MoS_2 powder exhibits narrow diffraction peaks while the commercial Sigma MoS_2 peaks are much wider (Supplementary Figure 26). We further note that the differences in peak height are due to texturing for the in-house CVT MoS_2 powder structure, even after grinding. The

low-defect density at the interfaces and the low oxygen content (see XPS Analysis in the Supplementary Information below) allow the lamellae to naturally stack together, creating a preference for orientation along the stacking direction.

Source powder samples for transmission electron microscopy (TEM) were prepared by suspending the powder in ethanol and drop casting on a lacey carbon support grid. Supplementary Figure 27a-c and S28a-c are HRTEM images showing structural similarity between the Sigma and CVT MoS_2 flakes under the same orientation as shown in the digital fast Fourier transform (FFT)-amplitude images in Supplementary Figure 27c and 28c. These images show similar lattice order, further suggesting high quality bulk powders at the atomic scale.

Overall, the starting bulk crystal source is an important initial consideration as it will contribute to the resulting exfoliated MoS_2 dimension, morphology, and defect density (such as lattice vacancies and oxidation). Furthermore, note that the efficacy of the exfoliation chemistry (e.g., the yield, flake size selective post-processing, colloidal stability, oxidative stability, and processability) is likewise expected to be dependent on the compositional purity of the starting source material due to percent oxidation and/or defect density.

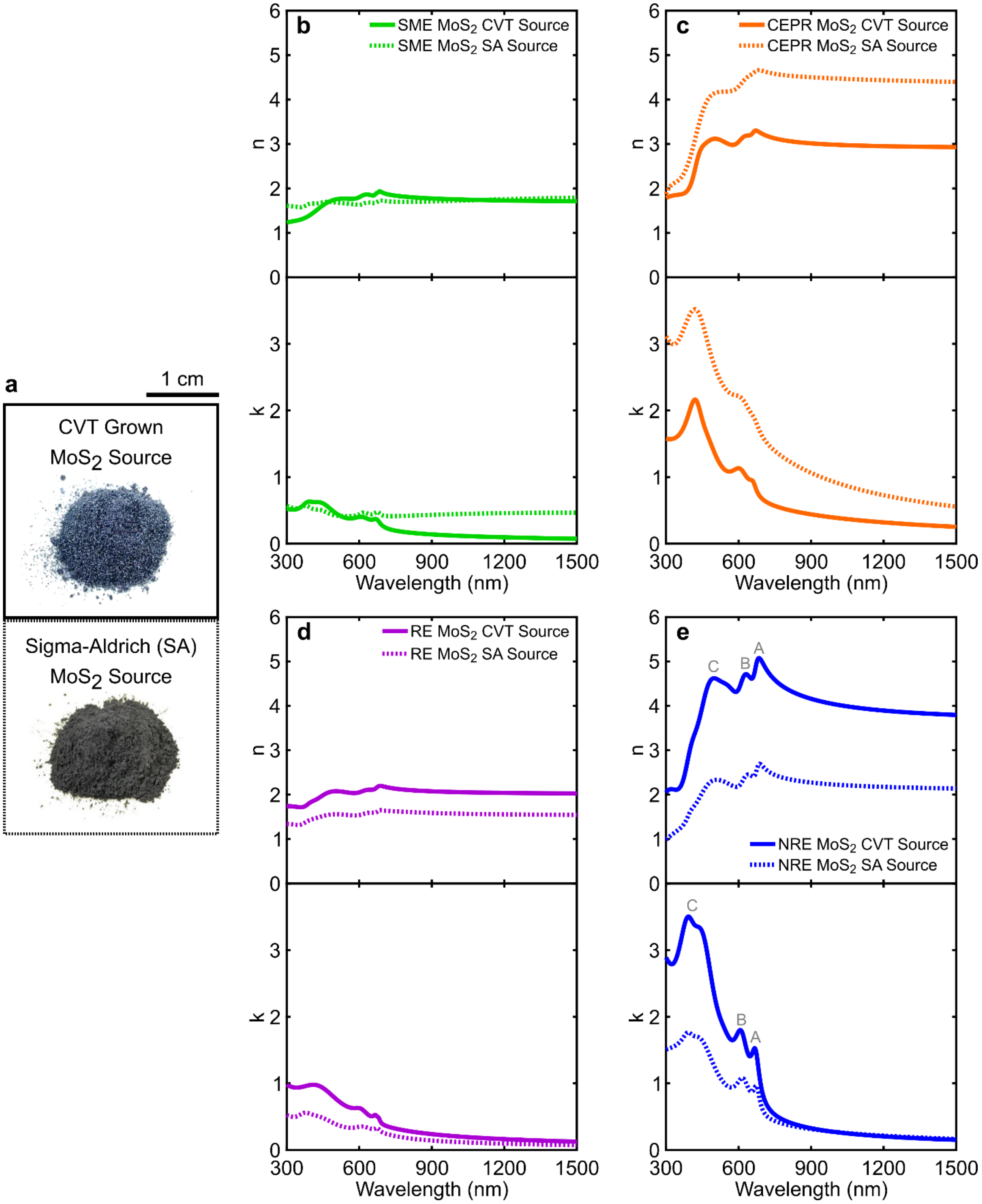

Supplementary Figure 23. Optical properties of exfoliated MoS_2 films from a commercial MoS_2 powder (Sigma-Aldrich, SA or Sigma) compared to our starting CVT MoS_2 powder used throughout this study. Powders are shown in (**a**). The optical property source powder comparison are shown in (**b**) for SME MoS_2, (**c**) for CEPR MoS_2, (**d**) RE MoS_2, and (**e**) NRE MoS_2.

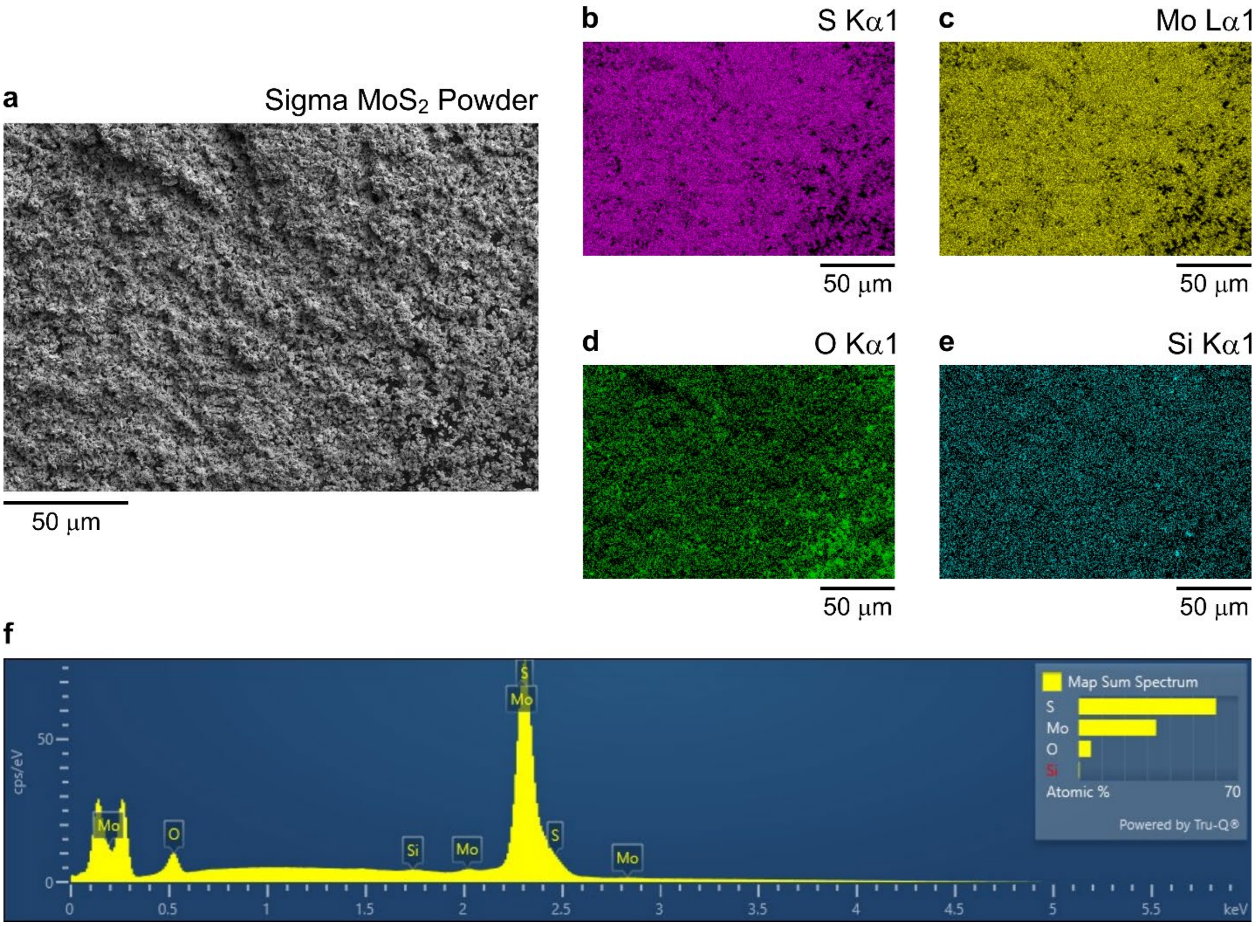

Supplementary Figure 24. SEM characterization of the Sigma-Aldrich MoS_2 powder. (**a**) SEM micrograph of Sigma powder - secondary electron image, (**b**-**e**) elemental maps generated by EDS analysis, and (**f**) the sum spectrum of X-ray counts over analysis area.

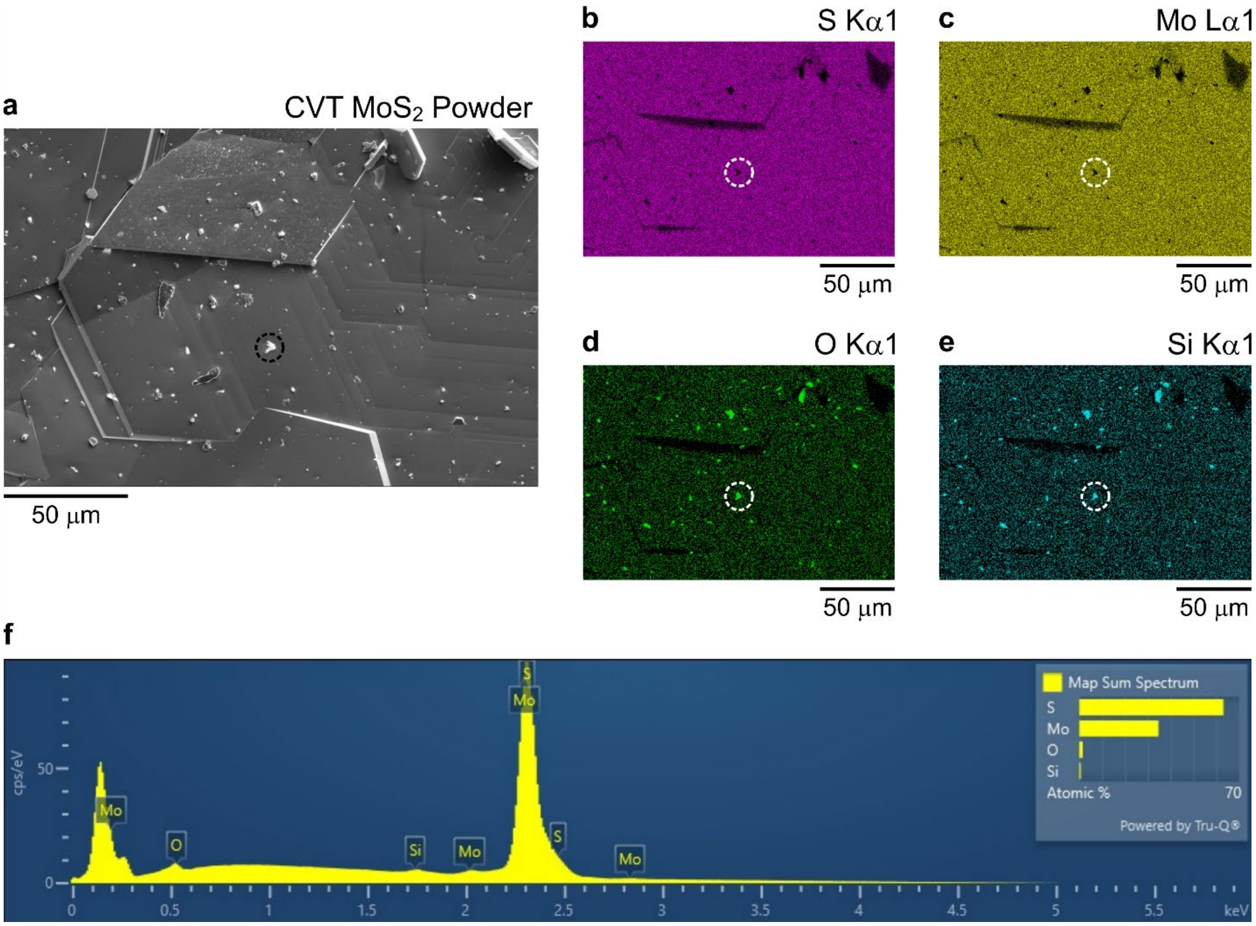

Supplementary Figure 25. SEM characterization of the CVT MoS_2 powder. (**a**) SEM micrograph of CVT MoS_2 powder - secondary electron image, (**b-e**) elemental maps generated by EDS analysis, and (**f**) sum spectrum of X-ray counts over analysis area. Note: the dashed circle in the micrograph and elemental maps indicate the same discrete particle with Si and O enrichment.

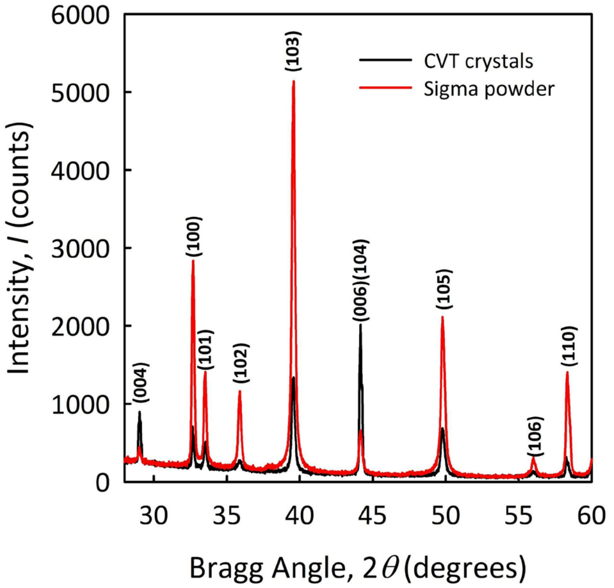

Supplementary Figure 26. Powder diffraction of two different MoS_2 starting source powders. CVT crystals grown in-house and commercial powder from Sigma-Aldrich. Relative peak height differences are due to the texturing exhibited by the in-house CVT samples due to the larger sized crystals intrinsic to this growth technique. The Sigma powder exhibits peak broadening in peaks associated with in-plane reflections while all reflections associated with the stacking direction are relatively narrow. This suggests that any defects are primarily due to in-plane disorder.

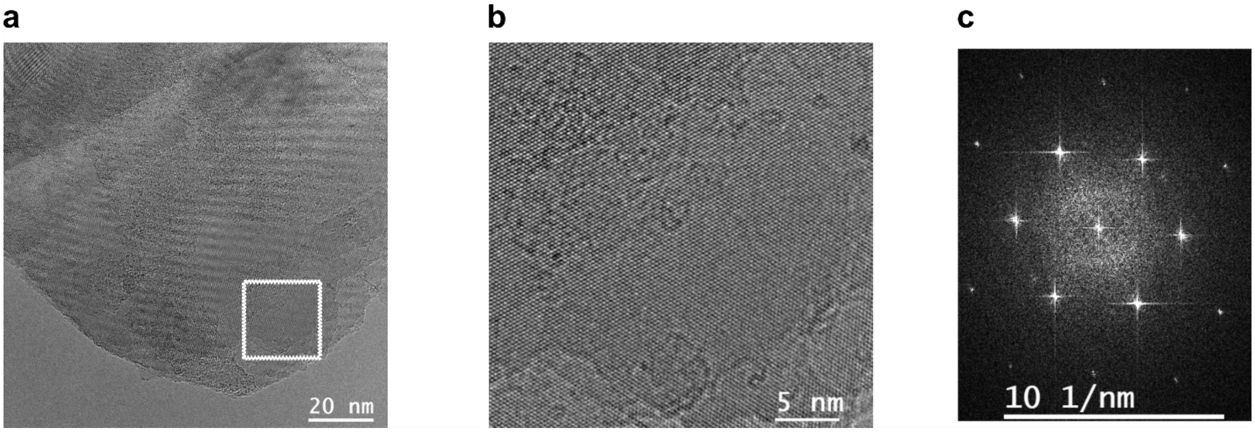

Supplementary Figure 27. TEM characterization of the Sigma MoS_2 powder. (**a**) bright-field TEM image of a flake suspended on lacey carbon support film, (**b**) magnified HRTEM image of the region marked in (**a**), and (**c**) digital fast Fourier transform (amplitude) image of (**b**).

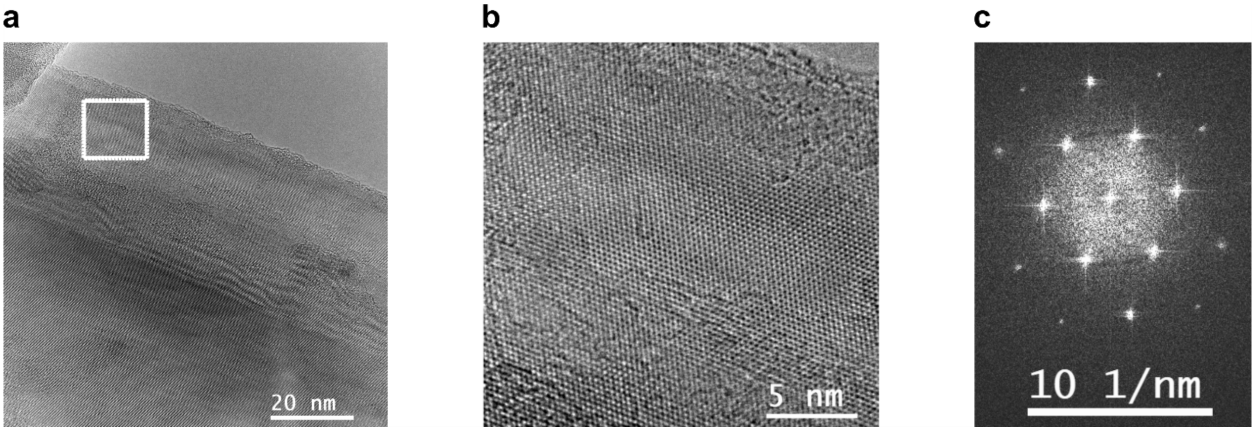

Supplementary Figure 28. TEM characterization CVT MoS_2 powder. (**a**) bright-field TEM image of a flake suspended in lacey carbon support film, (**b**) magnified HRTEM image of the region indicated in (**a**), and (**c**) digital fast Fourier transform (amplitude) image of (**b**).

Supplementary References

1. Mehmood, F. *et al.* Two-dimensional MoS_2 2H, 1T, and 1T′ crystalline phases with incorporated adatoms: theoretical investigation of electronic and optical properties. *Appl. Opt., AO* **60**, G232–G242 (2021).
2. Hilfiker, J. N. *et al.* Estimating Depolarization with the Jones Matrix Quality Factor. *Appl. Surf. Sci.* **421**, 494–499 (2017).
3. Busch, R. T. *et al.* Effective Optical Properties of Laterally Coalescing Monolayer MoS2. *J. Phys. Chem. Lett.* **13**, 5808–5814 (2022).
4. Macleod, A. The Quarterwave Stack: 1 Early History. *Bulletin, Society of Vacuum Coaters, Issue Summer* 22–27 (2012).
5. Garmire, E. Theory of quarter-wave-stack dielectric mirrors used in a thin fabry-perot filter. *Appl. Opt.* **42**, 5442–5449 (2003).
6. Zhang, X., Qiu, J., Li, X., Zhao, J. & Liu, L. Complex refractive indices measurements of polymers in visible and near-infrared bands. *Appl. Opt.* **59**, 2337–2344 (2020).
7. Stevenson, P. R. *et al.* Reversibly Tailoring Optical Constants of Monolayer Transition Metal Dichalcogenide MoS2 Films: Impact of Dopant-Induced Screening from Chemical Adsorbates and Mild Film Degradation. *ACS Photonics* **8**, 1705–1717 (2021).
8. Mouri, S., Miyauchi, Y. & Matsuda, K. Tunable photoluminescence of monolayer MoS_2 via chemical doping. *Nano Lett.* **13**, 5944–5948 (2013).
9. Zhang, X. *et al.* Diffusion-Controlled Epitaxy of Large Area Coalesced WSe2 Monolayers on Sapphire. *Nano Lett.* **18**, 1049–1056 (2018).
10. Kresse, G. & Furthmüller, J. Efficiency of ab-initio total energy calculations for metals and semiconductors using a plane-wave basis set. *Comput. Mater. Sci.* **6**, 15–50 (1996).

11. Kresse, G. & Joubert, D. From ultrasoft pseudopotentials to the projector augmented-wave method. *Phys. Rev. B Condens. Matter* **59**, 1758–1775 (1999).

12. Perdew, J. P., Burke, K. & Wang, Y. Generalized gradient approximation for the exchange-correlation hole of a many-electron system. *Physical Review B* vol. 54 16533–16539 Preprint at https://doi.org/10.1103/physrevb.54.16533 (1996).

13. Gajdoš, M., Hummer, K., Kresse, G., Furthmüller, J. & Bechstedt, F. Linear optical properties in the projector-augmented wave methodology. *Phys. Rev. B Condens. Matter* **73**, 045112 (2006).

14. Jawaid, A. M., Ritter, A. J. & Vaia, R. A. Mechanism for Redox Exfoliation of Layered Transition Metal Dichalcogenides. *Chem. Mater.* **32**, 6550–6565 (2020).

15. Schönfeld, B., Huang, J. J. & Moss, S. C. Anisotropic mean-square displacements (MSD) in single-crystals of 2H- and 3R-MoS2. *Acta Crystallogr. B* **39**, 404–407 (1983).
